# Early Transmission Dynamics, Spread, and Genomic Characterization of SARS-CoV-2 in Panama

**DOI:** 10.3201/eid2702.203767

**Published:** 2021-02

**Authors:** Danilo Franco, Claudia Gonzalez, Leyda E. Abrego, Jean-Paul Carrera, Yamilka Diaz, Yaset Caicedo, Ambar Moreno, Oris Chavarria, Jessica Gondola, Marlene Castillo, Elimelec Valdespino, Melissa Gaitán, Jose Martínez-Mandiche, Lizbeth Hayer, Pablo Gonzalez, Carmen Lange, Yadira Molto, Dalis Mojica, Ruben Ramos, Maria Mastelari, Lizbeth Cerezo, Lourdes Moreno, Christl A. Donnelly, Juan Miguel Pascale, Nuno Rodrigues Faria, Sandra Lopez-Verges, Alexander A. Martinez, on behalf of Gorgas COVID19 team and Panama COVID19 Laboratory Network.

**Affiliations:** Gorgas Memorial Institute of Health Studies, Panama City, Panama (D. Franco, C. Gonzalez, L.E. Abrego, J.-P. Carrera, Y. Diaz, A. Moreno, O. Chavarria, J. Gondola, M. Castillo, E. Valdespino, M. Gaitán, J. Martínez-Mandiche, D. Mojica, R. Ramos, J.M. Pascale, S. Lopez-Verges, A.A. Martinez);; Universidad de Panama, Panama City (D. Franco, C. Gonzalez, L.E. Abrego, J.M. Pascale, S. Lopez-Verges, A.A. Martinez);; Ministry of Health of Panama, Panama City (L. Hayer, P. Gonzalez, C. Lange, Y. Molto, M. Mastelari, L. Cerezo, L. Moreno);; University of Oxford, Oxford, UK (J.-P. Carrera, C.A. Donnelly, N.R. Faria);; Fundación Valle del Lili, Cali, Colombia (Y. Caicedo);; Imperial College, London, UK (C.A. Donnelly, N.R. Faria)

**Keywords:** Bayesian analysis, coronavirus disease, COVID-19 transmission, early cryptic transmission, epidemic dynamics, genomic diversity, outbreaks, Panama, respiratory infections, SARS-CoV-2, viruses

## Abstract

We report an epidemiologic analysis of 4,210 cases of infection with severe acute respiratory syndrome coronavirus 2 and genetic analysis of 313 new near-complete virus genomes in Panama during March 9–April 16, 2020. Although containment measures reduced R_0_ and R_t_, they did not interrupt virus spread in the country.

Coronavirus disease (COVID-19), caused by severe acute respiratory syndrome coronavirus 2 (SARS-CoV-2), was first reported in December 2019 in Wuhan, China (*1*,*2*). Of ≈23 million confirmed cases worldwide, as of October 20, 2020, a total of 28% (>6 million) had been reported in Latin America. SARS-CoV-2 was first reported in this region in São Paulo, Brazil, on February 25, 2020 (*3*). 

In Panama, the first confirmed COVID-19 case was reported on March 9, 2020. Although Panama rapidly implemented disease control strategies, it is among the countries in Latin America with the highest cumulative rates of incidence and death (*4*). To elucidate the transmission and spread of SARS-CoV-2 in the region, we analyzed epidemiologic surveillance data and newly generated genetic data from Panama.

## The Study

To perform molecular detection of SARS-CoV-2, the Panama Ministry of Health implemented a surveillance program on January 20, 2020. The National Committee on Bioethics of Research of Panama approved protocol EC-CNBI-202–04–46. 

We evaluated the early transmission dynamics of COVID-19 in Panama for the first 62 days of the epidemic (February 15–April 16, 2020) based on reported dates of symptom onset. We estimated the daily growth rate, doubling time, and basic (R_0_) and time-varying (R_t_) effective reproduction numbers. We performed genome amplification and sequencing according to ARTIC Network protocol (https://artic.network) for Illumina Sequencing (https://www.illumina.com) (*5*). Details of epidemic parameters, sequencing, and genome analysis are described in Appendix 2. 

**Figure 1:**
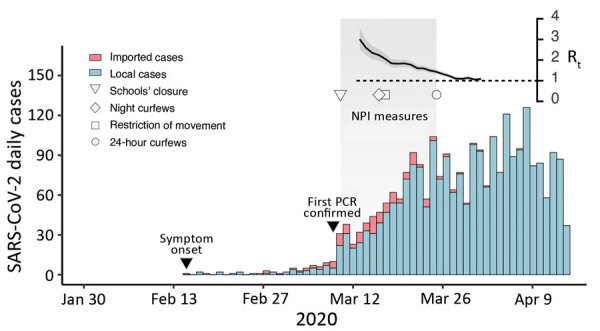
Epidemic curve of SARS-CoV-2 cases in Panama showing daily incidence of confirmed imported and local infections detected through April 16, 2020, with symptom onset during February 15–April 13, 2020. Gray shaded area indicates time period during which nonpharmaceutical interventions measures were initiated. Inset at top right shows the time-varying effective reproduction number (R_t_) for a time frame of 45 days (x-axis); dark gray shading indicates 95% CI, and dashed line indicates threshold value R_t_ = 1. SARS-CoV-2, severe acute respiratory syndrome coronavirus 2.

A total of 18,559 suspected cases of COVID-19 had been investigated in Panama by April 16. Of these, 4,210 (22.7%) patients tested positive for SARS-CoV-2 infection by qualitative reverse transcription PCR. The first confirmed case, on March 9, corresponded to a patient who had arrived in Panama from Spain on March 8 and had exhibited symptoms beginning on March 6. The first case not related to travel was confirmed after the death on March 7 of a patient in whom symptoms first appeared on February 22. Epidemiologic investigation showed that the date of onset of symptoms for the earliest local case related to that fatal case dates back to February 15, 2020 (Figure 1). In most locally detected cases, patients had mild disease symptoms (Appendix 2 Figure 1, panel A). 

**Figure 2:**
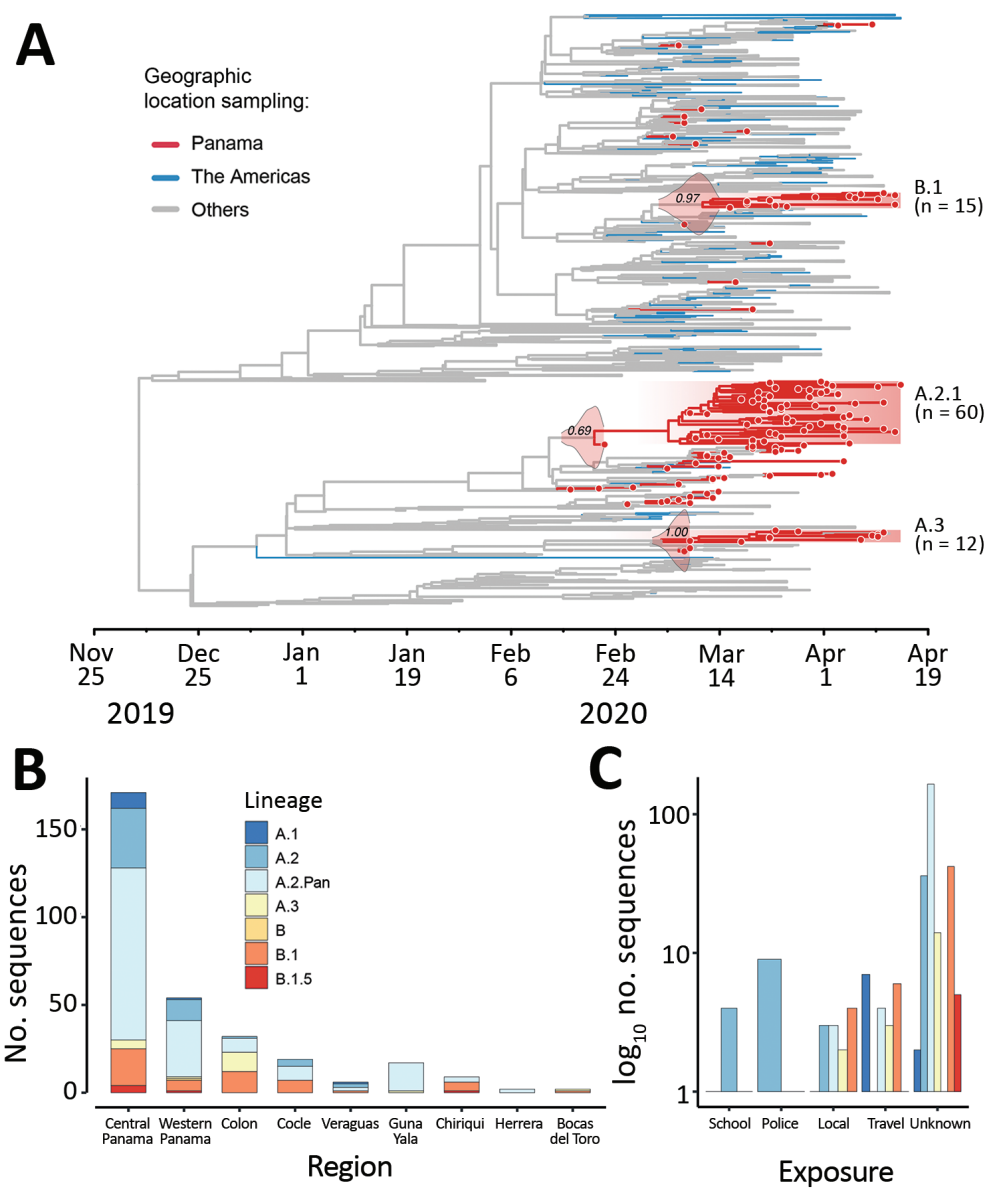
Genetic diversity of SARS-CoV-2 in Panama. A) Bayesian maximum clade credibility tree of 1,261 SARS-CoV-2 sequences: 133 from Panama; 492 from North or South America (443 genomes are from Brazil, 41 from the United States, 7 from Chile, 6 from Mexico, 3 from Argentina, 1 from Peru, and 1 from Canada); and 636 are from other locations. Posterior density estimates of time of the most recent common ancestor of each lineage with local transmission are shown in their branches. B) Distribution of lineages among regions in Panama. C) Distribution of lineages by channel of exposure detected by the surveillance system. SARS-CoV-2, severe acute respiratory syndrome coronavirus 2.

By April 16, a total of 341 patients had been hospitalized (77 at time of diagnosis confirmation) and 116 had died (31 by time of diagnosis confirmation) (Appendix 2 Figure 1, panels B, C). The highest proportion of confirmed cases was observed in the 20–59 year age group (Appendix 2 Figure 2, panel A). A higher proportion (55.3%) of patients tested were female, but among those with positive results, 1.45 times more were male (Appendix 2 Figure 2, panel B). A rapid growth rate of 0.13 cases/day (Appendix 2 Figure 3, panel A) and a short doubling time were observed during the early stages of the epidemic; doubling time increased over the study period (Appendix 2 Figure 3, panel B). We estimated an R_0_ for SARS-CoV-2 in Panama of 2.22 (95% CI 2.08–2.37). 

Panama was the 11th country in Latin America to report SARS-CoV-2 and implemented epidemic control strategies rapidly compared with other countries in the region (Appendix 2 Figure 4). After the first confirmed case (March 9), school closures were implemented within 1 day, social distancing measures within 6 days, and 24-hour stay-at-home curfew within 14 days. Over the course of the next 17 days, R_t_ dropped to 1.08 (95% Cl 1.00−1.17) (Appendix 2 Table 1, Figure 3, panel C). However, until April 16, Panama remained the country in Central America with the highest proportional number of cases and fatalities (Appendix 2 Figure 5). 

To determine the diversity of SARS-CoV-2 in Panama and Latin America, we generated SARS-CoV-2 genomes from 313 patients, representing 7.4% of the total confirmed cases by April 16, 2020 (Appendix 2 Figure 6, panel A). We obtained complete genome coverage for samples using reverse transcription PCR cycle threshold values <25 (Appendix 2 Figure 6, panel B) and found circulation of >10 virus lineages (Figure 2, panel A; Appendix 2 Figure 7) (*6*). The most frequently identified was A.2 (71.2%), followed by B.1 (16.7%) and A.1 (3.5%), in contrast to other studies in Latin America, where B-like lineages largely predominate (*7*,*8*). Lineages A.3, B, and B.1.5 were identified in 79 cases detected early on in the epidemic, 11 (13.9%) of the cases imported (Figure 2, panel A; Appendix 2 Figure 7). Lineage A.2 was found in 51 patients; 4 (7.8%) belonged to a cluster (Appendix 2 Table 2) from a school outbreak associated with the first detected local case and 9 (17.6%) were police officers (Figure 2, panel C). 

Phylogenetic analysis identified 3 main virus lineages (Figure 2). Lineage A.2.1/19B (n = 60; posterior support = 0.69; C12815T) comprised 54.3% of the sequenced cases in the study (Appendix 2 Figure 8, panel A); lineage B.1/20A (n = 15; posterior support = 0.97; G26143A) and lineage A.3/19B (n = 12; posterior support = 1.00; C3177T, T26729C) was third. Molecular clock estimates of the time to most recent common ancestor calculated from lineage A.2.1, made up just of cases with local transmission, placed the median time of mutation during February 19–March 9, 2020, just 2 weeks before the first COVID-19 case was confirmed, and in line with the time of onset of symptoms of the first case of local transmission (Figures 1, 2). 

Central and western Panama had more diverse lineage distributions (Figure 2, panel B). These regions encompass the capital and its surroundings, where more than 50% of the national population lives and the main international airport is located. Lineage A.2.1 was found in all regions across the country with no obvious spatial pattern; according to a global analysis of SARS-CoV-2 lineages (https://cov-lineages.org), this lineage is composed of sequences predominantly from Panama. We also found that the spike glycoprotein variants D614 and G614 (*9*,*10*) were cocirculating early in the epidemic among all the regions analyzed and were comprised of multiple lineages (Appendix 2 Figure 8, panel B), but the G614 variant potentially associated with infectivity (*9*) was detected in only 18.8% of the sequenced cases (Appendix 2 Figure 8, panel C). 

## Conclusions 

Epidemiologic evidence suggested cryptic circulation of SARS-CoV-2 in Panama with a probable introduction during early February. A high median transmission potential of SARS-CoV-2 was estimated at R_0_ = 2.22 (2.08–2.37), similar to estimates from China, Brazil, and Europe (*11*–*13*). R_t_ rapidly dropped to 1.08 after implementation of control strategies. 

Phylogenetic analysis detected circulation of >10 virus lineages, although the number of detected lineages could be underestimated because we did not sequence each positive case and there is a possibility of uncommon undetected lineages due to sample bias. Most of the lineages associated with imported cases (A.1, A.3, B, B.1, B.2.1) were detected and transmission controlled through active contact tracing. However, we detected early transmission of the lineage A.2.1/19B, which was introduced into the country >3 weeks before the first detected case. This lineage rapidly became widespread in Panama. 

We conjecture that efforts to identify early suspected cases, which focused mainly in symptomatic travelers returning from China, precluded the opportunity to detect earlier cases imported from Europe and the United States, where the virus was already circulating at that time (*11*,*14*,*15*). Moreover, undetected early transmission occurring before control measures were implemented could help to explain the widespread distribution of SARS-CoV-2 across Panama. 

Our findings on growth rates and R_t_ show that mitigation measures undertaken shortly after the first reported case in March helped to reduce virus transmission. Measures such as active contact tracing and isolation, social distancing, and quarantine targeted to regions where active transmission clusters are found will help to effectively control the spread of SARS-CoV-2 in Panama. 

Appendix 1 Members of the Gorgas COVID19 team and Panama COVID19 Laboratory Network, and laboratories contributing sequences from GISAID’s EpiFlu Database on which this research is based.

Appendix 2 Additional information for early transmission dynamics, spread, and genomic characterization of SARS-CoV-2 in Panama.
