# Supplementary material for: Early Transmission Dynamics, Spread, and Genomic Characterization of SARS-CoV-2 in Panama
Source: Emerg Infect Dis. 2021 Feb;27(2):612–5. doi: 10.3201/eid2702.203767 (PMC7853578; doi:10.3201/eid2702.203767)
Supplement: Appendix 2 — Additional information for early transmission dynamics, spread, and genomic characterization of SARS-CoV-2 in Panama. [file 20-3767-Techapp-s2.pdf]

Article DOI: https://doi.org/10.3201/eid2702.203767

# Early Transmission Dynamics, Spread, and Genomic Characterization of SARS-CoV-2 in Panama

## Appendix 2

## Methods

### Additional Details of COVID-19 Surveillance and Laboratory Diagnosis in Panama

The surveillance program for COVID-19 was implemented by the Panama Ministry of Health (MoH) on January 20, 2020. Suspected cases were actively sought at international airports using World Health Organization/Pan American Health Organization case definitions and recommendations (*1*). In this first stage, suspected cases were defined by symptoms and signs from influenza-like illness (ILI) and severe acute respiratory infections (SARI), as well as patients coming into Panama from China. On March 4, the list of countries from which travellers were monitored was expanded to include Italy, Iran, South Korea, and Japan. Travelers from these countries, confirmed cases, and their contacts were isolated in home or hotel quarantine for 14 days. Clinical evaluation and temperature measurement were done every day; if symptoms developed, nasopharyngeal or oropharyngeal swabs were taken by MoH and sent for laboratory diagnosis. For confirmed cases, contact tracing was performed by MoH.

The Gorgas Memorial Institute for Health Studies (GMI) is the national reference laboratory of Panama and is recognized as a World Health Organization National Influenza Center (*2*). The National Laboratory Network for Surveillance of Respiratory Viruses collects data from 16 sentinel sites through the national territory and 3 laboratories (Obaldia Hospital in Chiriqui province for eastern Panama, Chicho Fabrega Hospital in Veraguas province for Central Panama, and GMI in Panama City). The National COVID-19 Laboratory Network was built from the National Laboratory Network for Surveillance of Respiratory Viruses, but after increasing equipment capacity and training laboratory personnel intended to perform the molecular diagnosis of SARS-CoV-2, this network was extended to include more public health laboratories (from MoH as well as from the social security system and private hospitals).

Through April 16, 12 laboratories were part of the network; this was expanded to ≥20 laboratories for molecular diagnosis around the country.

Nasopharyngeal and oropharyngeal samples were collected in 503CS01 nasopharyngeal nylon swabs (Copan Diagnostics, https://www.fishersci.com) in 305C viral transport medium (Copan Diagnostics) to preserve the integrity of the viral particles. The collected samples were sent to GMI for laboratory confirmation beginning January 23 and to additional laboratories from the National COVID-19 Laboratory network beginning March 16. Samples were sent with a surveillance form with demographic, clinical, and contact tracing information (date of onset of symptoms, description of symptoms, age, sex, district of residence, district of employment, travel in or outside the country ≤15 days before symptom onset; if the person had contact with a positive patient: name and phone number of that person were also recorded).

The processing of SARS-CoV-2 suspected samples was performed in a BSL-2 facility with negative pressure. After viral inactivation, viral RNA was obtained by using QIAamp Viral Mini kit (QIAGEN, https://www.qiagen.com) according to the manufacturer's recommendations. During January 23–February 16, for SARS-CoV-2 suspected cases, the presence of viral RNA from coronavirus was detected using a generic reverse transcription PCR (RT-PCR) (*3*) and Sanger sequencing of the fragment as confirmation. Charité Institute's SARS-CoV-2–specific real time RT-PCR was implemented, using the E gene as screening and the RdRp gene of SARS-CoV-2 as confirmation (*4*), by using the One-Step RT-PCR kit AgPath-ID (Applied Biosystems, https://www.thermofisher.com) or the Invitrogen SuperScript III One-Step RT-qPCR (ThermoFisher, https://www.thermofisher.com) and the ABI 7500 Fast computer platform v1.4.0 System (Applied Biosystems). The fluorescence was read at the annealing/extension step and the threshold cycle ($C_t$) value for each PCR reaction was recorded. Samples with Ct values ≤40 were considered positive.

### Additional Information of Epidemiologic Investigation and Epidemic Parameters Estimation

Epidemiologic data on suspected cases and their contacts were recorded by physicians, using a standardized epidemiologic form for surveillance of respiratory viruses, at the airport, health facilities, or at home or hotel during quarantine, when nasopharyngeal and oropharyngeal swabs were taken. Data entry was independently undertaken by GMI and the laboratories from the National COVID-19 Laboratory network, and then checked by the National Department of

Epidemiology of MoH to confirm the accuracy of the information. A dataset of daily incidence based on the date of symptom onset was created, for samples reported through April 16 with the dates of first onset of symptoms during February 15–April 14. Data on delay in notification of patients was corrected by using the median of delay between onset of symptoms and report of confirmed cases. After the epidemic curve correction was done, the first 61 days of COVID-19 epidemic in Panama, February 15–April 13, were analyzed.

### General Description

Data on the demographics (age, sex, region) and clinical condition (ambulatory, for asymptomatic, presymptomatic, or mildly symptomatic outpatient patients; hospitalized; or fatal) of suspected patients were collected from MoH's respiratory virus surveillance sheet through the COVID-19 surveillance laboratory network. Data were evaluated until April 13 to correct reporting delay. We undertook several epidemiologic analyses.

We estimated the basic reproductive number ($R_0$) using the time series of confirmed cases with likelihood-based estimation using a branching process, following Poisson likelihood standards (*5*). A serial interval mean of 4.7 days, SD 2.9 was used for the estimation (*6*). The time-variant effective reproductive number ($R_t$) was estimated in a Bayesian framework because the cumulative number of cases reached 25 as described elsewhere (*6*), serial interval with 95% confidence intervals (95% Cl), using the EpiEstim package (https://cran.r-project.org) implemented in R (*5*).

### Epidemic Curve

The number of confirmed cases reported through April 15 reflects the number of cases per day using the date of symptom onset reported by the patient to the clinician or, when necessary, extrapolated from the date of sample receipt by GMI (*6*).

### Transmission Dynamics

To analyze local transmission dynamics through April 13, imported cases were removed from the epidemic curve. In addition, to avoid giving a false impression of decreasing transmissibility by a potential delay in the appearance and detection of cases because of the artificial drop in the epidemic curve from removing imported cases, the curve was corrected by removing the last 4 days of observations in the epidemic curve.

### Daily Growth Rate and Doubling Time

The daily growth rate was estimated using the formula $log(I) = r * t + b$, where $r$ is the incidence based on dates of symptom onset, $t$ is the length of time since onset, and $b$ is the intercept. The doubling time was calculated using $Dt = \frac{log(2)}{r}$ in an interval of 7.7 days. The growth rate with 95% confidence intervals was visualized using the Incidence package.

### Basic Reproductive Number

Daily reported incidence based on dates of symptom onset was used to estimate $R_0$ and $R_t$. To estimate $R_0$, several methods were explored, using serial intervals reported elsewhere (*6*), of mean 4.7 days, SD 2.9 days, according to gamma distribution (shape = 0.118, scale = 39.52). Early $R_0$ with likelihood-based estimation using a branching process follows Poisson likelihood standards described elsewhere (*5*). $R_0$ was estimated from data collected during February 28–March 24, 2020, to avoid variation due to implementation of control strategies.

### Effective Reproductive Number

We undertook approaches to estimate the time-varying effective reproductive number $R_t$, with a serial interval distribution described elsewhere (*6*), using a Bayesian framework. For this, the $R_t$ since the cumulative incidence reached 25 cases was obtained to reduce the coefficient of variation to 0.2. After 25 cases, the final time range analyzed included 31 days (March 13–April 13). $R_t$ with 95% confidence intervals (95% Cl), was estimated every 7 days using the EpiEstim package implemented in R (*5*).

### SARS-CoV-2 Genomic Characterization

To genetically characterize the SARS-CoV-2 strains introduced in Panama, as well as to analyze their distribution, samples were selected from areas where new cases were confirmed by laboratory during March 8–April 16, taking into account the cumulative number of cases for regions, as described elsewhere (*7*). In total, we selected 421 confirmed cases. Besides the clinical, epidemiologic, and demographic data, the $C_t$ values of the diagnostic RT-qPCR were also recorded (Appendix 2 Table 2).

Viral RNA extraction was performed and the RNA was transcribed to cDNA using the reverse transcription protocol by using Invitrogen SuperScript III First-Strand Synthesis System (ThermoFisher) with random hexamers and a pool of ARTIC reverse primers (https://artic.network) set to 50 μm. To amplify SARS-CoV-2–specific cDNA, a PCR reaction

was done by using Invitrogen Platinum Taq HiFi (ThermoFisher) with ARTIC Network protocol (*8*). PCR products were confirmed by agarose gel electrophoresis with evidence of a band of ≈450bp.

Amplicons generated were pooled and prepared for Illumina sequencing with the Nextera XT (https://www.illumina.com) library, according to the manufacturer's standard protocol. All samples were sequenced with MiSeq V2 (https://www.illumina.com) reagent kits for 500 cycles.

### Bioinformatics Analysis

The reads obtained were filtered with a minimum quality score of Q30 and 120 bp length, with Quasitools-hydra pipeline (https://github.com) (*9*) using the SARS-CoV-2 reference sequence MN908947.3. Variants were determined using a consensus based on a predetermined error rate of 0.0021, a minimum read depth of 10×, an allele count of 5, and a variant quality score of Q30. The average coverage obtained was 92.9%, IQR 0.05.

### Quality Control of Genome Consensus Sequences

A total of 365 PCR fragments were sequenced. To verify their quality for downstream analysis a quality control step was included; briefly, consensus sequences were filtered according to a genome coverage >75% and region-spanning nucleotide positions were masked with a python script (https://github.com) used elsewhere (*10*). After these steps, a total of 313 sequences were included in the phylogenetic analysis (Appendix 2 Figure 7). To maximize phylogenetic signal and reduce masked regions for Bayesian analysis (Figure 2, panel A), only sequences with >29,000 bp (~97% of coverage compared with MN908947.3 reference strain) were included (133 sequences).

### SARS-CoV-2 Global Dataset

The dataset used for Bayesian analysis was made up of 433 genomes from Brazil previously described (*7*), and genomes retrieved from gisaid.org; of these, 41 genomes were from the United States, 7 from Chile, 6 from Mexico, 3 from Argentina, 1 from Peru, 1 from Canada, and 636 from countries outside of the Americas (Appendix 2 Table 3), corresponding to a global subsampling of sequences including 1 genome per country per day (based on sampled collection day). This global dataset was retrieved on April 24, 2020. We sequenced genomes from Panama, Brazil, and from other parts of the world in the dataset used for this analysis.

### Phylogenetic Analysis of Panamanian SARS-CoV-2 Sequences

To perform the phylogenetic analysis, we adopted nomenclature described elsewhere (*11*), which was based on the dynamics of propagation of the virus according to the country of origin of the strain, with the objective of evaluating the lineage distribution in the country over time (*11*). The pangoLEARN pipeline, version 2020–08–29_3 (https://github.com) was used to assign lineage. To review the phylogenetic structure of SARS-CoV-2 virus in Panama, sequences were aligned using mafft v7.445 (https://mafft.cbrc.jp) (*12*), a high speed multiple sequence alignment program, and the maximum likelihood tree was inferred with IQTREE (http://www.iqtree.org) (*13*) using a substitution model (HKY + ⌈4 gamma variation) suggested previously (*7,14–16*) and visualized in FigTree 1.14 (http://tree.bio.ed.ac.uk) (Appendix 2 Figure 7).

### Temporal Signal of SARS-CoV-2 in Panama

To explore the temporal signal of SARS-CoV-2 in Panama, a Bayesian coalescent phylogeny was calculated using BEAST v.1.10.4 (https://beast.community) (*17*), using the same substitution model as in the maximum likelihood analysis, under an uncorrelated lognormal molecular clock, with a noninformative continuous-time Markov chain reference prior distribution (*18*). The exponential population growth tree model was implemented as in previous studies (*14,15*). The analysis was run for 250 million chains using BEAGLE v3 (https://github.com) (*19*) to enhance computational speed, writing to log every 25,000 chains. Convergence of the MCMC chains was inspected using Tracer v.1.7.1 (https://beast.community) (*20*). After removal of 10% burn-in, tree files were resampled using LogCombiner v.1.10.4 (https://beast.community) (*17*) to obtain a posterior sample of 1,000 dated phylogenetic trees. Maximum clade credibility summary trees were generated using TreeAnnotator v.1.10.4 (https://beast.community) (65). Time of the most recent common ancestor was calculated for each monophyletic lineage formed by sequenced with confirmed only local transmission from the same posterior tree distribution. Sequence mutations were annotated using the pipeline implemented in (*16*) and plotted with ggplot2 suite (https://rstudio.com) (*21*).

### Data and Materials Availability

### Control Measures in Latin America

Reports from the World Health Organization and the Organisation for Economic Co-operation and Development, and official communications from ministries of health were used to obtain data on control and mitigation strategies at the country level to reconstruct a timeline representing the strategies adopted by Latin American countries. Only if no official information was found, were press articles from local newspapers consulted.

### References


1. World Health Organization. Coronavirus disease (COVID-19) pandemic [cited 2020 August 26] https://www.who.int/emergencies/diseases/novel-coronavirus-2019

2. World Health Organization. National influenza centres. [cited 2020 Jun 9]. https://www.who.int/influenza/gisrs_laboratory/national_influenza_centres/list/en/index1.html

3. de Souza Luna LK, Heiser V, Regamey N, Panning M, Drexler JF, Mulangu S, et al. Generic detection of coronaviruses and differentiation at the prototype strain level by reverse transcription-PCR and nonfluorescent low-density microarray. J Clin Microbiol. 2007;45:1049–52. PubMed https://doi.org/10.1128/JCM.02426-06

4. Corman VM, Landt O, Kaiser M, Molenkamp R, Meijer A, Chu DK, et al. Detection of 2019 novel coronavirus (2019-nCoV) by real-time RT-PCR. Euro Surveill. 2020;25:2000045. PubMed https://doi.org/10.2807/1560-7917.ES.2020.25.3.2000045

5. Cori A, Ferguson NM, Fraser C, Cauchemez S. A new framework and software to estimate time-varying reproduction numbers during epidemics. Am J Epidemiol. 2013;178:1505–12. PubMed https://doi.org/10.1093/aje/kwt133

6. Nishiura H, Linton NM, Akhmetzhanov AR. Serial interval of novel coronavirus (COVID-19) infections. Int J Infect Dis. 2020;93:284–6. PubMed https://doi.org/10.1016/j.ijid.2020.02.060

7. Candido DS, Claro IM, de Jesus JG, Souza WM, Moreira FRR, Dellicour S, et al. Evolution and epidemic spread of SARS-CoV-2 in Brazil. Science. 2020;369:1255–60. PubMed https://doi.org/10.1126/science.abd2161

8. Quick J. nCoV-2019 sequencing protocol. 2020 [Cited 2020 November 22] https://dx.doi.org/10.17504/protocols.io.bibtkann

9. Taylor T, Lee ER, Nykoluk M, Enns E, Liang B, Capina R, et al. A MiSeq-HyDRA platform for enhanced HIV drug resistance genotyping and surveillance. Sci Rep. 2019;9:8970. PubMed https://doi.org/10.1038/s41598-019-45328-3

10. Hadfield J, Megill C, Bell SM, Huddleston J, Potter B, Callender C, et al. Nextstrain: real-time tracking of pathogen evolution. Bioinformatics. 2018;34:4121–3. PubMed https://doi.org/10.1093/bioinformatics/bty407

11. Rambaut A, Holmes EC, O'Toole Á, Hill V, McCrone JT, Ruis C, et al. A dynamic nomenclature proposal for SARS-CoV-2 lineages to assist genomic epidemiology. Nat Microbiol. 2020;5:1403–7. PubMed https://doi.org/10.1038/s41564-020-0770-5

12. Katoh K, Standley DM. MAFFT multiple sequence alignment software version 7: improvements in performance and usability. Mol Biol Evol. 2013;30:772–80. PubMed https://doi.org/10.1093/molbev/mst010

13. Nguyen LT, Schmidt HA, von Haeseler A, Minh BQ. IQ-TREE: a fast and effective stochastic algorithm for estimating maximum-likelihood phylogenies. Mol Biol Evol. 2015;32:268–74. PubMed https://doi.org/10.1093/molbev/msu300

14. Oude Munnink BB, Nieuwenhuijse DF, Stein M, O'Toole Á, Haverkate M, Mollers M, et al.; Dutch-Covid-19 response team. Rapid SARS-CoV-2 whole-genome sequencing and analysis for informed public health decision-making in the Netherlands. Nat Med. 2020;26:1405–10. Corrected and republished from: Nat Med. 2020;26:1802. PubMed https://doi.org/10.1038/s41591-020-0997-y

15. Nie Q, Li X, Chen W, Liu D, Chen Y, Li H, et al. Phylogenetic and phylodynamic analyses of SARS-CoV-2. Virus Res. 2020;287:198098. PubMed https://doi.org/10.1016/j.virusres.2020.198098

16. Lu J, du Plessis L, Liu Z, Hill V, Kang M, Lin H, et al. Genomic epidemiology of SARS-CoV-2 in Guangdong province, China. Cell. 2020;181:997–1003. PubMed https://doi.org/10.1016/j.cell.2020.04.023

17. Suchard MA, Lemey P, Baele G, Ayres DL, Drummond AJ, Rambaut A. Bayesian phylogenetic and phylodynamic data integration using BEAST 1.10. Virus Evol. 2018;4:vey016. PubMed https://doi.org/10.1093/ve/vey016

18. Ferreira MAR, Suchard MA. Bayesian analysis of elapsed times in continuous-time Markov chains. Can J Stat. 2008;36:355–68. https://doi.org/10.1002/cjs.5550360302

19. Ayres DL, Cummings MP, Baele G, Darling AE, Lewis PO, Swofford DL, et al. BEAGLE 3: improved performance, scaling, and usability for a high-performance computing library for statistical phylogenetics. Syst Biol. 2019;68:1052–61. PubMed https://doi.org/10.1093/sysbio/syz020

20. Rambaut A, Drummond AJ, Xie D, Baele G, Suchard MA. Posterior summarization in Bayesian phylogenetics using Tracer 1.7. Syst Biol. 2018;67:901–4. PubMed https://doi.org/10.1093/sysbio/syy032

21. Wickham H. ggplot2: elegant graphics for data analysis. 2nd ed. New York: Springer; 2016.

**Appendix 2 Table 1.** Estimates of the reproductive number ($R_t$) over time.

| Observations | t_start | t_end | Mean(R) | Std(R) | Quantile 0.025 (R) | Quantile 0.975 (R) | Dates |
|---|---|---|---|---|---|---|---|
| 1 | 2 | 9 | 3.01 | 0.25 | 2.54 | 3.52 | 3/13/2020 |
| 2 | 3 | 10 | 2.59 | 0.20 | 2.22 | 2.99 | 3/14/2020 |
| 3 | 4 | 11 | 2.35 | 0.16 | 2.04 | 2.68 | 3/15/2020 |
| 4 | 5 | 12 | 2.24 | 0.14 | 1.97 | 2.53 | 3/16/2020 |
| 5 | 6 | 13 | 2.06 | 0.12 | 1.82 | 2.30 | 3/17/2020 |
| 6 | 7 | 14 | 1.84 | 0.11 | 1.64 | 2.05 | 3/18/2020 |
| 7 | 8 | 15 | 1.82 | 0.10 | 1.64 | 2.02 | 3/19/2020 |
| 8 | 9 | 16 | 1.84 | 0.09 | 1.67 | 2.02 | 3/20/2020 |
| 9 | 10 | 17 | 1.78 | 0.08 | 1.62 | 1.94 | 3/21/2020 |
| 10 | 11 | 18 | 1.61 | 0.07 | 1.47 | 1.76 | 3/22/2020 |
| 11 | 12 | 19 | 1.60 | 0.07 | 1.47 | 1.73 | 3/23/2020 |
| 12 | 13 | 20 | 1.49 | 0.06 | 1.37 | 1.62 | 3/24/2020 |
| 13 | 14 | 21 | 1.43 | 0.06 | 1.32 | 1.55 | 3/25/2020 |
| 14 | 15 | 22 | 1.32 | 0.05 | 1.22 | 1.43 | 3/26/2020 |
| 15 | 16 | 23 | 1.23 | 0.05 | 1.14 | 1.33 | 3/27/2020 |
| 16 | 17 | 24 | 1.11 | 0.05 | 1.02 | 1.20 | 3/28/2020 |
| 17 | 18 | 25 | 1.09 | 0.04 | 1.01 | 1.18 | 3/29/2020 |
| 18 | 19 | 26 | 1.14 | 0.04 | 1.05 | 1.23 | 3/30/2020 |
| 19 | 20 | 27 | 1.05 | 0.04 | 0.97 | 1.14 | 3/31/2020 |
| 20 | 21 | 28 | 1.08 | 0.04 | 1.00 | 1.17 | 4/1/2020 |

**Appendix 2 Table 2.** Characteristic and demographic information of sequenced samples in the study.

| Case | Epi week | Sex | Age, y | Region | ID | GISAID Accession # | Date symptom onset | Date results received | Epi cluster | Epiclusterlink | Type of exposition | Epi link | Exposition description | Lineage autocolor |
|---|---|---|---|---|---|---|---|---|---|---|---|---|---|---|
| 1 | 10 | F | 40 | Panamá Metro | 328677 | EPI_ISL_415152 | 2020-03-06 | 2020-03-09 | 1 | travel | imported | travel | Spain | B.1.5 |
| 3 | 10 | F | 29 | Panamá Metro | 328688 | EPI_ISL_496603 | 2020-03-04 | 2020-03-09 | 2 | travel | imported | travel | USA | A.3 |
| 4 | 10 | F | 43 | Panamá Metro | 328706 | EPI_ISL_496604 | 2020-03-05 | 2020-03-09 | 4 | travel | imported | travel | Spain | A.2 |
| 5 | 9 | M | 64 | Panamá Norte | 328709 | EPI_ISL_496605 | 2020-02-28 | 2020-03-09 | 3 | school | local | school | NA | A.2 |
| 6 | 8 | M | 49 | Panamá Metro | 328710 | EPI_ISL_496606 | 2020-02-20 | 2020-03-09 | 3 | school | local | unknown | NA | A.2.1 |
| 7 | 8 | F | 58 | Panamá Metro | 328719 | EPI_ISL_496607 | 2020-02-22 | 2020-03-10 | 3 | school | local | unknown | NA | A.2 |
| 8 | 10 | F | 35 | Panamá Metro | 328721 | EPI_ISL_496608 | 2020-03-09 | 2020-03-10 | 5 | travel | imported | travel | USA | A.1 |
| 9 | 8 | F | 57 | Panamá Norte | 328723 | EPI_ISL_496609 | 2020-02-17 | 2020-03-10 | 3 | school | local | school | NA | A.2 |
| 11 | 11 | M | 60 | San Miguelito | 328726 | EPI_ISL_496610 | 2020-03-08 | 2020-03-10 | 6 | travel | imported | travel | USA | A.3 |
| 12 | 10 | M | 61 | Panamá Metro | 328733 | EPI_ISL_496611 | 2020-03-07 | 2020-03-10 | NA | unknown | imported | travel | USA | A.1 |
| 14 | 11 | M | 38 | Panamá Metro | 328774 | EPI_ISL_496612 | 2020-03-10 | 2020-03-10 | 4 | travel | local | contact | Travel | A.2.1 |
| 15 | 10 | M | 31 | Panamá Metro | 328844 | EPI_ISL_496613 | 2020-03-07 | 2020-03-11 | NA | unknown | imported | travel | Spain | B.1.5 |
| 16 | 11 | F | 28 | Panamá Metro | 328848 | EPI_ISL_496614 | 2020-03-08 | 2020-03-11 | NA | unknown | imported | travel | Europe | B.1 |
| 17 | 11 | M | 34 | Panamá Metro | 328927 | EPI_ISL_496615 | 2020-02-15 | 2020-03-11 | 1 | travel | imported | travel | Spain | B.1 |
| 18 | 11 | F | 44 | Panamá Metro | 328933 | EPI_ISL_496616 | 2020-03-09 | 2020-03-11 | 6 | travel | imported | travel | Puerto Rico | A.3 |
| 19 | 9 | F | 37 | San Miguelito | 328941 | EPI_ISL_496617 | 2020-02-27 | 2020-03-11 | 5 | travel | imported | travel | USA | A.1 |
| 20 | 10 | M | 43 | San Miguelito | 328944 | EPI_ISL_496618 | 2020-03-04 | 2020-03-11 | 5 | travel | imported | travel | USA | A.1 |
| 23 | 11 | F | 70 | Panamá Metro | 328971 | EPI_ISL_496619 | 2020-03-09 | 2020-03-11 | 15 | travel | imported | travel | USA | A.1 |
| 24 | 11 | M | 41 | Panamá Norte | 328972 | EPI_ISL_496620 | 2020-03-08 | 2020-03-11 | 3 | school | local | contact | School | A.1 |
| 26 | 10 | M | 40 | Panamá Norte | 328980 | EPI_ISL_496621 | 2020-03-07 | 2020-03-11 | 3 | school | local | contact | School | A.2 |
| 27 | 11 | F | 10 | Panamá Norte | 328981 | EPI_ISL_496622 | 2020-03-10 | 2020-03-11 | 7 | travel | imported | travel | France, Italy | A.2.1 |
| 30 | 11 | M | 42 | Panamá Este | 329064 | EPI_ISL_496623 | 2020-03-09 | 2020-03-12 | 8 | police | local | police | NA | A.2 |
| 32 | 11 | M | 42 | Panamá Metro | 329108 | EPI_ISL_496624 | 2020-03-10 | 2020-03-12 | | unknown | imported | travel | Spain | B.1.5 |
| 33 | 11 | F | 48 | Panamá Oeste | 329117 | EPI_ISL_496625 | 2020-03-08 | 2020-03-12 | 1 | travel | imported | travel | Spain, Switzerland | B.1 |
| 35 | 11 | F | 38 | Panamá Metro | 329198 | EPI_ISL_496626 | 2020-03-08 | 2020-03-12 | 9 | travel | imported | travel | Germany | B.1 |
| 36 | 10 | F | 13 | Panamá Metro | 329230 | EPI_ISL_496627 | 2020-03-05 | 2020-03-12 | 10 | travel | imported | travel | USA | A.1 |
| 40 | 11 | F | 44 | Panamá Metro | 329377 | EPI_ISL_496628 | 2020-03-13 | 2020-03-13 | NA | unknown | local | unknown | NA | B |
| 42 | 11 | F | 45 | Panamá Oeste | 329388 | EPI_ISL_496629 | 2020-03-11 | 2020-03-13 | 1 | travel | local | contact | Travel | B.1 |
| 44 | 11 | F | 60 | Panamá Norte | 329446 | EPI_ISL_496630 | 2020-03-12 | 2020-03-13 | 1 | travel | local | contact | Health | A.2.1 |
| 45 | 11 | F | 43 | Colón | 329536 | EPI_ISL_496631 | 2020-03-09 | 2020-03-14 | 26 | local | local | contact | Health | A.3 |
| 52 | 11 | F | 50 | Panamá Oeste | 329546 | EPI_ISL_496632 | 2020-03-12 | 2020-03-14 | 25 | local | local | contact | Health | A.2 |
| 53 | 11 | F | 40 | Panamá Oeste | 329547 | EPI_ISL_496633 | 2020-03-13 | 2020-03-14 | 5 | travel | local | contact | Travel | A.1 |
| 55 | 11 | M | 63 | Panamá Metro | 329560 | EPI_ISL_496634 | 2020-03-12 | 2020-03-14 | 10 | travel | imported | travel | USA | A.1 |
| 59 | 11 | F | 48 | Panamá Metro | 329576 | EPI_ISL_496635 | 2020-03-09 | 2020-03-15 | NA | unknown | local | school | NA | A.2 |
| 61 | 11 | M | 49 | Panamá Oeste | 329593 | EPI_ISL_496636 | 2020-03-13 | 2020-03-15 | 8 | police | local | police | NA | A.2 |
| 62 | 11 | M | 54 | Panamá Oeste | 329628 | EPI_ISL_496637 | 2020-03-11 | 2020-03-15 | 8 | police | local | police | NA | A.2 |
| 68 | 11 | M | 41 | Panamá Oeste | 329653 | EPI_ISL_496638 | 2020-03-10 | 2020-03-15 | 17 | local | local | unknown | NA | A.2.1 |
| 69 | 11 | F | 41 | Panamá Metro | 329655 | EPI_ISL_496639 | 2020-03-11 | 2020-03-15 | NA | unknown | local | school | NA | A.2.1 |
| 70 | 11 | M | 48 | Panamá Metro | 329667 | EPI_ISL_496640 | 2020-03-14 | 2020-03-16 | 8 | police | local | police | NA | A.2 |
| 71 | 11 | F | 51 | San Miguelito | 329676 | EPI_ISL_496641 | 2020-03-12 | 2020-03-16 | 8 | police | local | police | NA | A.2 |
| 73 | 10 | F | 34 | Veraguas | 329682 | EPI_ISL_496642 | 2020-03-06 | 2020-03-16 | 14 | local | local | unknown | NA | A.2 |
| 74 | 11 | M | 36 | Panamá Oeste | 329694 | EPI_ISL_496643 | 2020-03-10 | 2020-03-16 | 20 | local | local | unknown | NA | A.1 |
| 75 | 12 | M | 48 | Panamá Metro | 329700 | EPI_ISL_496644 | 2020-03-16 | 2020-03-16 | 8 | police | local | police | NA | A.2 |
| 77 | 12 | F | 30 | Panamá Oeste | 329718 | EPI_ISL_496645 | 2020-03-15 | 2020-03-16 | 8 | police | local | police | NA | A.2 |
| 78 | 11 | M | 62 | Panamá Metro | 329728 | EPI_ISL_496646 | 2020-03-14 | 2020-03-16 | NA | unknown | local | unknown | NA | A.1 |
| 80 | 11 | M | 54 | Panamá Metro | 329734 | EPI_ISL_496648 | 2020-03-14 | 2020-03-16 | NA | unknown | imported | travel | USA | B.1 |

| Case | Epi week | Sex | Age, y | Region | ID | GISAID Accession # | Date symptom onset | Date results received | Epi cluster | Epiclusterlink | Type of exposition | Epi link | Exposition description | Lineage autocolor |
|---|---|---|---|---|---|---|---|---|---|---|---|---|---|---|
| 82 | 11 | M | 45 | Panamá Metro | 329752 | EPI_ISL_496649 | 2020-03-14 | 2020-03-16 | NA | unknown | local | contact | Health | A.2.1 |
| 88 | 11 | F | 28 | San Miguelito | 329844 | EPI_ISL_496650 | 2020-03-12 | 2020-03-16 | NA | unknown | local | unknown | NA | A.2 |
| 91 | 11 | M | 57 | Panamá Metro | 329862 | EPI_ISL_496651 | 2020-03-14 | 2020-03-16 | 23 | travel | local | contact | Travel | B.1 |
| 92 | 11 | F | 29 | Panamá Norte | 329868 | EPI_ISL_496652 | 2020-03-13 | 2020-03-16 | NA | unknown | local | unknown | NA | A.2 |
| 93 | 10 | M | 45 | Veraguas | 329877 | EPI_ISL_496653 | 2020-03-06 | 2020-03-16 | 18 | local | local | unknown | NA | B.1 |
| 94 | 11 | F | 61 | Veraguas | 329879 | EPI_ISL_496654 | 2020-03-14 | 2020-03-16 | 14 | local | local | unknown | NA | A.2 |
| 95 | 10 | M | 34 | Panamá Metro | 329893 | EPI_ISL_496655 | 2020-03-09 | 2020-03-17 | NA | unknown | local | unknown | NA | A.2.1 |
| 97 | 11 | F | 70 | Panamá Metro | 329916 | EPI_ISL_496656 | 2020-03-13 | 2020-03-17 | NA | unknown | local | unknown | NA | A.2.1 |
| 112 | 12 | M | 66 | Panamá Oeste | 330057 | EPI_ISL_496657 | 2020-03-15 | 2020-03-17 | NA | unknown | local | unknown | NA | A.2.1 |
| 116 | 11 | M | 55 | Panamá Metro | 330089 | EPI_ISL_496658 | 2020-03-13 | 2020-03-17 | 8 | police | local | police | NA | A.2 |
| 121 | 12 | M | 53 | Panamá Metro | 330130 | EPI_ISL_496659 | 2020-03-15 | 2020-03-17 | 2 | travel | local | contact | Health | A.2.1 |
| 129 | 11 | M | 42 | Panamá Oeste | 330208 | EPI_ISL_496660 | 2020-03-10 | 2020-03-18 | NA | unknown | local | unknown | NA | A.2.1 |
| 134 | 11 | M | 52 | Panamá Metro | 330286 | EPI_ISL_496661 | 2020-03-09 | 2020-03-18 | 18 | local | local | contact | MP | B.1 |
| 138 | 11 | M | 40 | Panamá Norte | 330339 | EPI_ISL_496662 | 2020-03-09 | 2020-03-18 | 8 | police | local | police | NA | B.1 |
| 157 | 11 | M | 30 | Panamá Oeste | 330413 | EPI_ISL_496663 | 2020-03-13 | 2020-03-18 | NA | unknown | local | police | NA | A.2 |
| 163 | 11 | M | 40 | Panamá Oeste | 330449 | EPI_ISL_496664 | 2020-03-13 | 2020-03-18 | NA | unknown | local | unknown | NA | A.2 |
| 170 | 11 | F | 37 | Panamá Este | 330490 | EPI_ISL_496665 | 2020-03-19 | 2020-03-19 | NA | unknown | local | unknown | NA | A.2 |
| 175 | 11 | M | 46 | Coclé | 330547 | EPI_ISL_496666 | 2020-03-14 | 2020-03-19 | NA | unknown | local | police | NA | A.2 |
| 176 | 11 | M | 37 | Panamá Oeste | 330553 | EPI_ISL_496667 | 2020-03-11 | 2020-03-19 | 20 | local | local | unknown | NA | A.2.1 |
| 177 | 12 | M | 14 | Panamá Metro | 330558 | EPI_ISL_496668 | 2020-03-18 | 2020-03-19 | 11 | travel | local | contact | Travel | B.1 |
| 194 | 12 | F | 46 | Colón | 330671 | EPI_ISL_496669 | 2020-03-18 | 2020-03-19 | 26 | local | local | contact | Health | A.3 |
| 197 | 11 | M | 63 | Panamá Metro | 330722 | EPI_ISL_496670 | 2020-03-13 | 2020-03-19 | NA | unknown | local | police | NA | A.2 |
| 202 | 11 | M | 46 | Panamá Oeste | 330754 | EPI_ISL_496671 | 2020-03-12 | 2020-03-19 | NA | unknown | local | unknown | NA | A.2.1 |
| 207 | 11 | F | 53 | Colón | 330771 | EPI_ISL_496672 | 2020-03-12 | 2020-03-19 | NA | unknown | local | unknown | NA | A.2.1 |
| 208 | 10 | M | 54 | Panamá Metro | 330775 | EPI_ISL_496673 | 2020-03-01 | 2020-03-19 | NA | unknown | local | unknown | NA | A.3 |
| 211 | 12 | F | 49 | Panamá Norte | 330779 | EPI_ISL_496674 | 2020-03-18 | 2020-03-19 | 19 | local | local | contact | Health | B.1 |
| 217 | 11 | M | 46 | Panamá Oeste | 330795 | EPI_ISL_496675 | 2020-03-14 | 2020-03-19 | NA | unknown | local | unknown | NA | B.1 |
| 223 | 11 | M | 41 | Panamá Metro | 330817 | EPI_ISL_496676 | 2020-03-10 | 2020-03-20 | NA | unknown | local | contact | MP | B.1 |
| 239 | 12 | F | 49 | Panamá Norte | 330961 | EPI_ISL_496677 | 2020-03-19 | 2020-03-20 | NA | unknown | local | unknown | NA | A.2.1 |
| 243 | 12 | F | 38 | Panamá Norte | 331025 | EPI_ISL_496678 | 2020-03-18 | 2020-03-20 | NA | unknown | local | unknown | NA | A.2 |
| 245 | 12 | F | 59 | Panamá Metro | 331050 | EPI_ISL_496679 | 2020-03-19 | 2020-03-20 | 28 | local | local | contact | Health | A.2.1 |
| 251 | 12 | M | 46 | Panamá Oeste | 331074 | EPI_ISL_496680 | 2020-03-18 | 2020-03-20 | NA | unknown | local | contact | Health | A.2.1 |
| 259 | 10 | F | 42 | Panamá Oeste | 331186 | EPI_ISL_496681 | 2020-03-06 | 2020-03-20 | NA | unknown | local | unknown | NA | A.2.1 |
| 262 | 11 | M | 43 | Panamá Norte | 331254 | EPI_ISL_496682 | 2020-03-11 | 2020-03-20 | NA | unknown | local | unknown | NA | A.2.1 |
| 270 | 12 | F | 10 | Panamá Metro | 331336 | EPI_ISL_496683 | 2020-03-20 | 2020-03-21 | 30 | local | local | contact | Travel | B.1 |
| 291 | 12 | F | 2 | Panamá Este | 331499 | EPI_ISL_496684 | 2020-03-17 | 2020-03-21 | NA | unknown | local | contact | Police | A.2.1 |
| 297 | 10 | M | 49 | Panamá Este | 331516 | EPI_ISL_496685 | 2020-03-21 | 2020-03-21 | NA | unknown | local | unknown | NA | A.2.1 |
| 303 | 12 | F | 13 | Panamá Metro | 331540 | EPI_ISL_496686 | 2020-03-19 | 2020-03-22 | NA | unknown | local | unknown | NA | A.2 |
| 311 | 12 | M | 34 | Chiriqui | 331578 | EPI_ISL_496687 | 2020-03-19 | 2020-03-22 | NA | unknown | local | contact | MP | B.1 |
| 312 | 12 | M | 46 | Chiriqui | 331580 | EPI_ISL_496688 | 2020-03-19 | 2020-03-22 | NA | unknown | local | contact | MP | B.1 |
| 314 | 12 | F | 64 | Panamá Metro | 331596 | EPI_ISL_496689 | 2020-03-17 | 2020-03-22 | NA | unknown | local | unknown | NA | B.1 |
| 340 | 12 | F | 45 | Panamá Metro | 331701 | EPI_ISL_496690 | 2020-03-18 | 2020-03-22 | NA | unknown | local | unknown | NA | A.2.1 |
| 344 | 12 | F | 95 | Panamá Metro | 331715 | EPI_ISL_496691 | 2020-03-20 | 2020-03-22 | NA | unknown | local | unknown | NA | A.2 |
| 366 | 12 | F | 15 | Panamá Metro | 331789 | EPI_ISL_496692 | 2020-03-21 | 2020-03-23 | NA | unknown | local | unknown | NA | A.2.1 |
| 371 | 10 | M | 48 | Panamá Este | 331797 | EPI_ISL_496693 | 2020-03-20 | 2020-03-23 | NA | unknown | local | unknown | NA | A.2 |
| 379 | 12 | M | 49 | Panamá Norte | 331836 | EPI_ISL_496694 | 2020-03-18 | 2020-03-23 | NA | unknown | local | unknown | NA | A.2.1 |
| 380 | 12 | M | 30 | Panamá Oeste | 331837 | EPI_ISL_496695 | 2020-03-19 | 2020-03-23 | NA | unknown | local | contact | MP | B.1 |
| 387 | 12 | M | 55 | San Miguelito | 331872 | EPI_ISL_496696 | 2020-03-16 | 2020-03-23 | NA | unknown | local | unknown | NA | A.3 |

| Case | Epi week | Sex | Age, y | Region | ID | GISAID Accession # | Date symptom onset | Date results received | Epi cluster | Epiclusterlink | Type of exposition | Epi link | Exposition description | Lineage autocolor |
|---|---|---|---|---|---|---|---|---|---|---|---|---|---|---|
| 391 | 12 | F | 29 | Panamá Este | 331920 | EPI_ISL_496697 | 2020-03-21 | 2020-03-23 | NA | unknown | local | unknown | NA | A.2.1 |
| 395 | 12 | F | 58 | Panamá Oeste | 331943 | EPI_ISL_496698 | 2020-03-20 | 2020-03-23 | NA | unknown | local | unknown | NA | A.2.1 |
| 397 | 12 | M | 53 | Panamá Metro | 331954 | EPI_ISL_496699 | 2020-03-20 | 2020-03-23 | NA | unknown | local | unknown | NA | A.2 |
| 405 | 13 | F | 17 | Panamá Metro | 331997 | EPI_ISL_496700 | 2020-03-22 | 2020-03-23 | NA | unknown | local | unknown | NA | A.2.1 |
| 407 | 12 | M | 86 | San Miguelito | 332013 | EPI_ISL_496701 | 2020-03-19 | 2020-03-23 | NA | unknown | local | unknown | NA | A.2.1 |
| 410 | 12 | M | 40 | Panamá Metro | 332033 | EPI_ISL_496702 | 2020-03-20 | 2020-03-23 | NA | unknown | local | unknown | NA | B.1.5 |
| 422 | 13 | M | 52 | Panamá Metro | 332089 | EPI_ISL_496703 | 2020-03-22 | 2020-03-23 | NA | unknown | local | unknown | NA | A.2.1 |
| 438 | 12 | M | 15 | Panamá Norte | 332231 | EPI_ISL_496704 | 2020-03-18 | 2020-03-23 | NA | unknown | local | unknown | NA | A.2.1 |
| 442 | 11 | M | 31 | Coclé | 332238 | EPI_ISL_496705 | 2020-03-14 | 2020-03-23 | NA | unknown | local | unknown | NA | A.2.1 |
| 445 | 13 | M | 49 | Guna Yala | 332252 | EPI_ISL_496706 | 2020-03-23 | 2020-03-24 | NA | unknown | local | unknown | NA | A.3 |
| 446 | 12 | M | 73 | Panamá Metro | 332254 | EPI_ISL_496707 | 2020-03-16 | 2020-03-24 | NA | unknown | local | unknown | NA | B.1 |
| 456 | 12 | F | 46 | Panamá Norte | 332298 | EPI_ISL_496708 | 2020-03-20 | 2020-03-24 | NA | unknown | local | unknown | NA | A.2.1 |
| 459 | 13 | M | 46 | Panamá Oeste | 332311 | EPI_ISL_496709 | 2020-03-22 | 2020-03-24 | NA | unknown | local | unknown | NA | A.2.1 |
| 472 | 13 | F | 60 | Panamá Metro | 332352 | EPI_ISL_496710 | 2020-03-23 | 2020-03-24 | NA | unknown | local | unknown | NA | A.2.1 |
| 481 | 12 | M | 40 | Panamá Metro | 332389 | EPI_ISL_496711 | 2020-03-19 | 2020-03-24 | NA | unknown | local | unknown | NA | A.2.1 |
| 500 | 12 | M | 60 | Panamá Metro | 332469 | EPI_ISL_496712 | 2020-03-22 | 2020-03-24 | NA | unknown | local | unknown | NA | A.2.1 |
| 511 | 13 | M | 55 | Panamá Oeste | 332513 | EPI_ISL_496713 | 2020-03-23 | 2020-03-24 | NA | unknown | local | unknown | NA | A.2.1 |
| 524 | 12 | F | 60 | Panamá Metro | 332575 | EPI_ISL_496714 | 2020-03-17 | 2020-03-24 | NA | unknown | local | unknown | NA | A.2.1 |
| 534 | 13 | M | 25 | Chiriqui | 332629 | EPI_ISL_496715 | 2020-03-24 | 2020-03-24 | NA | unknown | local | police | NA | B.1 |
| 549 | 13 | M | 39 | Panamá Metro | 332688 | EPI_ISL_496716 | 2020-03-23 | 2020-03-25 | NA | unknown | local | unknown | NA | A.2.1 |
| 551 | 13 | F | 70 | Panamá Metro | 332692 | EPI_ISL_496717 | 2020-03-23 | 2020-03-25 | NA | unknown | local | unknown | NA | A.2.1 |
| 554 | 12 | F | 39 | Panamá Este | 332702 | EPI_ISL_496718 | 2020-03-15 | 2020-03-25 | NA | unknown | local | unknown | NA | A.2.1 |
| 559 | 13 | M | 26 | Veraguas | 332718 | EPI_ISL_496719 | 2020-03-23 | 2020-03-25 | NA | unknown | local | police | NA | A.2.1 |
| 567 | 12 | F | 48 | Coclé | 332759 | EPI_ISL_496720 | 2020-03-20 | 2020-03-25 | NA | unknown | imported | travel | USA | A.2 |
| 568 | A | F | 22 | San Miguelito | 332762 | EPI_ISL_496721 | 2020-03-20 | 2020-03-25 | NA | unknown | local | unknown | NA | A.2.1 |
| 572 | 13 | F | 73 | Panamá Metro | 332791 | EPI_ISL_496722 | 2020-03-22 | 2020-03-25 | NA | unknown | local | unknown | NA | A.2.1 |
| 575 | 12 | M | 31 | Panamá Este | 332800 | EPI_ISL_496723 | 2020-03-20 | 2020-03-25 | NA | unknown | local | unknown | NA | A.2.1 |
| 577 | 13 | F | 35 | Panamá Metro | 332810 | EPI_ISL_496724 | 2020-03-23 | 2020-03-25 | NA | unknown | local | unknown | NA | A.2 |
| 582 | 13 | F | 59 | Panamá Metro | 332822 | EPI_ISL_496725 | 2020-03-25 | 2020-03-25 | NA | unknown | local | unknown | NA | A.2 |
| 583 | 12 | F | 44 | Panamá Metro | 332833 | EPI_ISL_496726 | 2020-03-25 | 2020-03-25 | NA | unknown | local | unknown | NA | A.2.1 |
| 587 | 13 | M | 52 | San Miguelito | 332856 | EPI_ISL_496727 | 2020-03-22 | 2020-03-25 | NA | unknown | local | unknown | NA | A.2.1 |
| 588 | 13 | F | 69 | San Miguelito | 332857 | EPI_ISL_496728 | 2020-03-24 | 2020-03-25 | NA | unknown | local | unknown | NA | A.2.1 |
| 596 | 13 | M | 54 | Panamá Metro | 332875 | EPI_ISL_496729 | 2020-03-22 | 2020-03-25 | NA | unknown | local | unknown | NA | A.2.1 |
| 623 | 13 | F | 7 | San Miguelito | 333048 | EPI_ISL_496730 | 2020-03-24 | 2020-03-25 | NA | unknown | local | unknown | NA | A.2 |
| 627 | 12 | F | 49 | San Miguelito | 333063 | EPI_ISL_496731 | 2020-03-21 | 2020-03-25 | NA | unknown | local | unknown | NA | A.2.1 |
| 657 | 13 | M | 25 | San Miguelito | 333156 | EPI_ISL_496732 | 2020-03-24 | 2020-03-26 | NA | unknown | local | unknown | NA | A.2 |
| 664 | 13 | M | 49 | Panamá Metro | 333177 | EPI_ISL_496733 | 2020-03-23 | 2020-03-26 | NA | unknown | local | unknown | NA | A.2.1 |
| 666 | 12 | F | 47 | Colón | 333181 | EPI_ISL_496734 | 2020-03-19 | 2020-03-26 | NA | unknown | local | unknown | NA | A.2.1 |
| 678 | 13 | F | 30 | Panamá Este | 333242 | EPI_ISL_496735 | 2020-03-25 | 2020-03-26 | NA | unknown | local | unknown | NA | A.2.1 |
| 687 | 12 | F | 71 | Panamá Metro | 333271 | EPI_ISL_496736 | 2020-02-23 | 2020-03-26 | NA | unknown | local | unknown | NA | A.2.1 |
| 714 | 13 | F | 24 | San Miguelito | 333338 | EPI_ISL_496737 | 2020-03-25 | 2020-03-26 | NA | unknown | local | unknown | NA | A.2.1 |
| 725 | 12 | M | 66 | Guna Yala | 333376 | EPI_ISL_496738 | 2020-03-16 | 2020-03-26 | NA | unknown | local | unknown | NA | A.2.1 |
| 734 | 12 | M | 45 | Coclé | 333393 | EPI_ISL_496739 | 2020-03-18 | 2020-03-26 | NA | unknown | local | unknown | NA | A.2 |
| 735 | 13 | F | 40 | San Miguelito | 333400 | EPI_ISL_496740 | 2020-03-23 | 2020-03-26 | NA | unknown | local | unknown | NA | A.2.1 |
| 740 | 13 | M | 66 | Panamá Oeste | 333427 | EPI_ISL_496741 | 2020-03-25 | 2020-03-26 | NA | unknown | local | contact | Health | A.2.1 |
| 742 | 13 | M | 36 | Panamá Oeste | 333430 | EPI_ISL_496742 | 2020-03-23 | 2020-03-26 | NA | unknown | local | contact | Health | A.2.1 |
| 753 | 13 | M | 77 | Panamá Metro | 333467 | EPI_ISL_496743 | 2020-03-23 | 2020-03-26 | NA | unknown | local | unknown | NA | B.1 |
| 758 | 13 | M | 65 | San Miguelito | 333487 | EPI_ISL_496744 | 2020-03-23 | 2020-03-26 | NA | unknown | local | contact | Family | B.1 |

| Case | Epi week | Sex | Age, y | Region | ID | GISAID Accession # | Date symptom onset | Date results received | Epi cluster | Epiclusterlink | Type of exposition | Epi link | Exposition description | Lineage autocolor |
|---|---|---|---|---|---|---|---|---|---|---|---|---|---|---|
| 765 | 13 | M | 29 | San Miguelito | 333517 | EPI_ISL_496745 | 2020-03-27 | 2020-03-27 | NA | unknown | local | unknown | NA | A.2.1 |
| 775 | 13 | M | 28 | Chiriqui | 333564 | EPI_ISL_496746 | 2020-03-26 | 2020-03-27 | NA | unknown | local | police | NA | B.1 |
| 777 | 12 | M | 45 | Coclé | 333567 | EPI_ISL_496747 | 2020-03-18 | 2020-03-27 | NA | unknown | local | contact | Police | B.1 |
| 778 | 12 | M | 42 | Coclé | 333568 | EPI_ISL_496748 | 2020-03-16 | 2020-03-27 | NA | unknown | local | contact | Police | B.1 |
| 809 | 13 | M | 48 | Guna Yala | 333668 | EPI_ISL_496749 | 2020-03-26 | 2020-03-27 | NA | unknown | local | unknown | NA | A.2.1 |
| 815 | 13 | F | 33 | Panamá Este | 333679 | EPI_ISL_496750 | 2020-03-24 | 2020-03-27 | NA | unknown | local | unknown | NA | A.2.1 |
| 817 | 13 | F | 58 | Panamá Metro | 333681 | EPI_ISL_496751 | 2020-03-23 | 2020-03-27 | NA | unknown | local | unknown | NA | A.2.1 |
| 818 | 13 | F | 49 | Barco Anclado en Amador | 333685 | EPI_ISL_496752 | 2020-03-24 | 2020-03-27 | NA | unknown | local | unknown | NA | A.2.1 |
| 821 | 13 | M | 19 | Colón | 333694 | EPI_ISL_496753 | 2020-03-23 | 2020-03-27 | NA | unknown | local | unknown | NA | A.3 |
| 827 | 12 | M | 29 | Colón | 333706 | EPI_ISL_496754 | 2020-03-20 | 2020-03-27 | NA | unknown | local | unknown | NA | A.2.1 |
| 853 | 13 | F | 86 | Panamá Metro | 333825 | EPI_ISL_496755 | 2020-03-26 | 2020-03-27 | NA | unknown | local | unknown | NA | A.2.1 |
| 881 | 12 | F | 29 | Colón | 333937 | EPI_ISL_496756 | 1900-01-00 | 2020-03-28 | NA | unknown | local | unknown | NA | A.3 |
| 883 | 12 | M | 39 | Colón | 333949 | EPI_ISL_496757 | 2020-03-27 | 2020-03-28 | NA | unknown | local | unknown | NA | A.2.1 |
| 894 | 13 | M | 49 | Panamá Metro | 334000 | EPI_ISL_496758 | 2020-03-23 | 2020-03-28 | NA | unknown | local | unknown | NA | B.1 |
| 903 | 13 | M | 48 | Chiriqui | 334032 | EPI_ISL_496759 | 2020-03-22 | 2020-03-28 | NA | unknown | local | police | NA | B.1 |
| 904 | 13 | M | 41 | Panamá Oeste | 334034 | EPI_ISL_496760 | 2020-03-26 | 2020-03-28 | NA | unknown | local | police | NA | B.1 |
| 905 | 13 | M | 40 | Bocas Del Toro | 334035 | EPI_ISL_496761 | 2020-03-24 | 2020-03-28 | NA | unknown | local | contact | MP | B.1 |
| 906 | 12 | M | 37 | Coclé | 334036 | EPI_ISL_496762 | 2020-03-25 | 2020-03-28 | NA | unknown | local | police | NA | B.1 |
| 907 | 13 | M | 22 | Panamá Este | 334038 | EPI_ISL_496763 | 2020-03-24 | 2020-03-28 | NA | unknown | local | police | NA | B.1 |
| 922 | A | M | 61 | Guna Yala | 334102 | EPI_ISL_496764 | 2020-03-21 | 2020-03-29 | NA | unknown | local | unknown | NA | A.2.1 |
| 930 | 13 | F | 35 | Panamá Oeste | 334115 | EPI_ISL_496765 | 2020-03-26 | 2020-03-29 | NA | unknown | local | unknown | NA | A.2.1 |
| 932 | 13 | F | 99 | Panamá Oeste | 334117 | EPI_ISL_496766 | 2020-03-19 | 2020-03-29 | NA | unknown | local | unknown | NA | B.1.5 |
| 937 | 13 | F | 40 | Colón | 334144 | EPI_ISL_496767 | 2020-03-24 | 2020-03-29 | NA | unknown | local | contact | Family | A.3 |
| 960 | - | F | 79 | Panamá Metro | 334239 | EPI_ISL_496768 | 2020-03-29 | 2020-03-29 | NA | unknown | local | unknown | NA | A.2.1 |
| 962 | - | M | 45 | Panamá Norte | 334245 | EPI_ISL_496769 | 2020-03-23 | 2020-03-29 | NA | unknown | local | unknown | NA | A.2 |
| 964 | 14 | M | 65 | Guna Yala | 334256 | EPI_ISL_496770 | 2020-03-26 | 2020-03-29 | NA | unknown | local | unknown | NA | A.2.1 |
| 969 | 13 | M | 26 | Panamá Oeste | 334277 | EPI_ISL_496771 | 2020-03-29 | 2020-03-30 | NA | unknown | local | unknown | NA | A.2.1 |
| 970 | 13 | M | 19 | Herrera | 334280 | EPI_ISL_496772 | 2020-03-28 | 2020-03-30 | NA | unknown | local | unknown | NA | A.2.1 |
| 973 | 13 | M | 27 | San Miguelito | 334283 | EPI_ISL_496773 | 2020-03-27 | 2020-03-30 | NA | unknown | local | unknown | NA | A.2.1 |
| 986 |  | F | 47 | Panamá Oeste | 334333 | EPI_ISL_496774 | 2020-03-25 | 2020-03-30 | NA | unknown | local | unknown | NA | A.2 |
| 1006 | 13 | F | 37 | San Miguelito | 334389 | EPI_ISL_496775 | 2020-03-29 | 2020-03-30 | NA | unknown | local | unknown | NA | A.2 |
| 1007 | 13 | M |  | San Miguelito | 334390 | EPI_ISL_496776 | 2020-03-30 | 2020-03-30 | NA | unknown | local | unknown | NA | A.2 |
| 1010 | A | M | 27 | Panamá Metro | 334396 | EPI_ISL_496777 | 2020-03-27 | 2020-03-30 | NA | unknown | local | unknown | NA | A.2.1 |
| 1013 | 13 | F | 60 | Panamá Oeste | 334400 | EPI_ISL_496778 | 2020-03-30 | 2020-03-30 | NA | unknown | local | unknown | NA | A.2.1 |
| 1031 |  | M | 29 | Panamá Oeste | 334440 | EPI_ISL_496779 | 2020-03-30 | 2020-03-30 | NA | unknown | local | contact | Health | A.2.1 |
| 1040 | 14 | F | 41 | Colón | 334456 | EPI_ISL_496780 | 2020-03-20 | 2020-03-30 | NA | unknown | local | unknown | NA | B.1 |
| 1041 | 13 | M | 46 | Panamá Metro | 334468 | EPI_ISL_496781 | 2020-03-30 | 2020-03-30 | NA | unknown | local | unknown | NA | A.2.1 |
| 1043 | 14 | M | 36 | Panamá Metro | 334475 | EPI_ISL_496782 | 2020-03-27 | 2020-03-30 | NA | unknown | local | unknown | NA | A.2.1 |
| 1068 | 14 | F | 33 | Coclé | 334554 | EPI_ISL_496783 | 2020-03-25 | 2020-03-30 | NA | unknown | local | unknown | NA | A.2.1 |
| 1069 | 14 | M | 43 | Colón | 334559 | EPI_ISL_496784 | 2020-03-22 | 2020-03-30 | NA | unknown | local | unknown | NA | A.2 |
| 1078 | 13 | F | 34 | San Miguelito | 334598 | EPI_ISL_496785 | 2020-03-22 | 2020-03-30 | NA | unknown | local | unknown | NA | A.2.1 |
| 1083 | 14 | F | 55 | San Miguelito | 334610 | EPI_ISL_496786 | 2020-03-29 | 2020-03-31 | NA | unknown | local | unknown | NA | A.2 |
| 1084 | 14 | M | 23 | Panamá Metro | 334611 | EPI_ISL_496787 | 2020-03-29 | 2020-03-31 | NA | unknown | local | unknown | NA | A.2.1 |
| 1087 | 13 | M | 34 | Panamá Norte | 334618 | EPI_ISL_496788 | 2020-03-27 | 2020-03-31 | NA | unknown | local | unknown | NA | A.2.1 |
| 1092 | 14 | M | 33 | Coclé | 334647 | EPI_ISL_496789 | 2020-03-29 | 2020-03-31 | NA | unknown | local | contact | MP | B.1 |
| 1095 | 14 | M | 41 | Coclé | 334655 | EPI_ISL_496790 | 2020-03-30 | 2020-03-31 | NA | unknown | local | unknown | NA | A.2.1 |
| 1098 | 14 | M | 4m | Panamá Oeste | 334658 | EPI_ISL_496791 | 2020-03-31 | 2020-03-31 | NA | unknown | local | unknown | NA | A.2.1 |

| Case | Epi week | Sex | Age, y | Region | ID | GISAID Accession # | Date symptom onset | Date results received | Epi cluster | Epiclusterlink | Type of exposition | Epi link | Exposition description | Lineage autocolor |
|---|---|---|---|---|---|---|---|---|---|---|---|---|---|---|
| 1105 | 13 | F | 55 | Panamá Metro | 334687 | EPI_ISL_496792 | 2020-03-26 | 2020-03-31 | NA | unknown | local | unknown | NA | A.2.1 |
| 1128 | 14 | M | 49 | Panamá Este | 334779 | EPI_ISL_496793 | 2020-03-29 | 2020-03-31 | NA | unknown | local | unknown | NA | A.2.1 |
| 1145 | | F | | Coclé | 334835 | EPI_ISL_496794 | 2020-03-31 | 2020-03-31 | NA | unknown | local | unknown | NA | B.1 |
| 1148 | 14 | M | 48 | Panamá Oeste | 334845 | EPI_ISL_496795 | 2020-03-30 | 2020-03-31 | NA | unknown | local | unknown | NA | A.2.1 |
| 1155 | 13 | M | 48 | Panamá Norte | 334868 | EPI_ISL_496796 | 2020-03-24 | 2020-03-31 | NA | unknown | local | unknown | NA | A.2.1 |
| 1159 | | F | 67 | Panamá Metro | 334898 | EPI_ISL_496797 | 2020-03-31 | 2020-03-31 | NA | unknown | local | unknown | NA | A.2.1 |
| 1161 | | M | 38 | Panamá Norte | 334913 | EPI_ISL_496798 | 2020-03-31 | 2020-03-31 | NA | unknown | local | unknown | NA | A.2.1 |
| 1167 | 13 | M | 28 | San Miguelito | 334998 | EPI_ISL_496799 | 2020-04-01 | 2020-04-01 | NA | unknown | local | unknown | NA | A.2.1 |
| 1169 | 14 | F | 25 | Panamá Oeste | 335002 | EPI_ISL_496800 | 2020-04-01 | 2020-04-01 | NA | unknown | local | unknown | NA | A.2.1 |
| 1171 | 14 | M | 30 | Coclé | 335013 | EPI_ISL_496801 | 2020-04-01 | 2020-04-01 | NA | unknown | local | unknown | NA | A.2 |
| 1184 | 14 | M | 37 | Panamá Norte | 335067 | EPI_ISL_496802 | 2020-03-31 | 2020-04-01 | NA | unknown | local | unknown | NA | B.1 |
| 1188 | 14 | F | 23 | Panamá Metro | 335090 | EPI_ISL_496803 | 2020-03-30 | 2020-04-01 | NA | unknown | local | unknown | NA | A.2 |
| 1190 | 13 | M | 18 | Panamá Este | 335097 | EPI_ISL_496804 | 2020-03-28 | 2020-04-01 | NA | unknown | local | unknown | NA | A.2.1 |
| 1191 | 14 | F | 18 | Panamá Este | 335099 | EPI_ISL_496805 | 2020-03-29 | 2020-04-01 | NA | unknown | local | unknown | NA | A.2.1 |
| 1197 | 14 | M | 28 | Panamá Oeste | 335119 | EPI_ISL_496806 | 2020-03-31 | 2020-04-01 | NA | unknown | local | unknown | NA | A.2.1 |
| 1208 | | F | 41 | Panamá Metro | 335156 | EPI_ISL_496807 | 2020-03-30 | 2020-04-01 | NA | unknown | local | unknown | NA | A.2.1 |
| 1216 | 14 | F | 9 | Panamá Metro | 335166 | EPI_ISL_496808 | 2020-03-31 | 2020-04-01 | NA | unknown | local | unknown | NA | A.2.1 |
| 1219 | 14 | F | 29 | Panamá Norte | 335182 | EPI_ISL_496809 | 2020-03-28 | 2020-04-01 | NA | unknown | local | unknown | NA | A.2 |
| 1255 | 14 | M | 41 | Veraguas | 335382 | EPI_ISL_496810 | 31-03-2020 | 2020-04-02 | NA | unknown | local | unknown | NA | A.2.1 |
| 1262 | 14 | F | 42 | Panamá Oeste | 335403 | EPI_ISL_496811 | NA | 2020-04-02 | NA | unknown | local | unknown | NA | A.2.1 |
| 1263 | 13 | M | 44 | Colón | 335406 | EPI_ISL_496812 | NA | 2020-04-02 | NA | unknown | local | unknown | NA | A.2.1 |
| 1267 | 13 | M | 27 | Panamá Este | 335473 | EPI_ISL_496813 | 2020-03-26 | 2020-04-02 | NA | unknown | local | unknown | NA | A.2.1 |
| 1268 | 14 | M | 89 | Panamá Metro | 335488 | EPI_ISL_496814 | 2020-03-30 | 2020-04-02 | NA | unknown | local | unknown | NA | B.1 |
| 1279 | 14 | M | 40 | Panamá Oeste | 335546 | EPI_ISL_496815 | NA | 2020-04-02 | 8 | police | local | unknown | NA | A.2 |
| 1284 | 14 | M | 33 | Coclé | 335568 | EPI_ISL_496816 | 2020-04-02 | 2020-04-02 | NA | unknown | local | unknown | NA | A.2.1 |
| 1285 | 14 | M | 45 | Panamá Oeste | 335572 | EPI_ISL_496817 | 2020-03-29 | 2020-04-02 | NA | unknown | local | unknown | NA | A.2.1 |
| 1302 | 13 | F | 19 | Panamá Norte | 335631 | EPI_ISL_496818 | NA | 2020-04-02 | NA | unknown | local | unknown | NA | A.2.1 |
| 1320 | 14 | M | 25 | Panamá Metro | 335723 | EPI_ISL_496819 | 2020-04-02 | 2020-04-03 | NA | unknown | local | unknown | NA | A.2.1 |
| 1342 | 13 | F | 61 | Panamá Metro | 335800 | EPI_ISL_496820 | 2020-03-28 | 2020-04-03 | NA | unknown | local | contact | Family | A.2.1 |
| 1348 | 14 | M | 16 | Panamá Oeste | 335859 | EPI_ISL_496821 | 2020-03-30 | 2020-04-03 | NA | unknown | local | unknown | NA | A.2.1 |
| 1349 | 14 | F | 44 | Panamá Oeste | 335862 | EPI_ISL_496822 | 2020-04-02 | 2020-04-03 | NA | unknown | local | contact | Family | A.2.1 |
| 1356 | 14 | M | 20 | Chiriqui | 335877 | EPI_ISL_496823 | 2020-04-01 | 2020-04-03 | NA | unknown | local | unknown | NA | A.2.1 |
| 1360 | 14 | M | 23 | Herrera | 335885 | EPI_ISL_496824 | 2020-04-01 | 2020-04-03 | NA | unknown | local | unknown | NA | A.2.1 |
| 1364 | 14 | M | 30 | Coclé | 335903 | EPI_ISL_496825 | 2020-04-01 | 2020-04-03 | NA | unknown | local | contact | Family | A.2.1 |
| 1367 | 14 | M | 24 | Panamá Este | 335922 | EPI_ISL_496826 | NA | 2020-04-03 | NA | unknown | local | contact | Family | A.2.1 |
| 1368 | 14 | F | 38 | Panamá Metro | 335925 | EPI_ISL_496827 | 2020-03-31 | 2020-04-03 | NA | unknown | local | unknown | NA | A.2 |
| 1370 | 14 | M | 61 | Guna Yala | 335931 | EPI_ISL_496828 | 2020-04-02 | 2020-04-03 | NA | unknown | local | unknown | NA | A.2.1 |
| 1375 | 14 | F | 54 | Panamá Oeste | 335941 | EPI_ISL_496829 | 01/04/2020 | 2020-04-03 | NA | unknown | local | unknown | NA | A.2 |
| 1378 | 14 | F | 31 | Panamá Metro | 335944 | EPI_ISL_496830 | 2020-04-01 | 2020-04-03 | NA | unknown | local | unknown | NA | A.2.1 |
| 1379 | 14 | M | 43 | Panamá Metro | 335945 | EPI_ISL_496831 | 2020-03-29 | 2020-04-03 | NA | unknown | local | unknown | NA | A.2.1 |
| 1385 | 14 | F | 36 | Panamá Norte | 335967 | EPI_ISL_496832 | 2020-04-03 | 2020-04-03 | NA | unknown | local | unknown | NA | A.2.1 |
| 1395 | 14 | F | 26 | Panamá Oeste | 336008 | EPI_ISL_496833 | 2020-03-30 | 2020-04-03 | NA | unknown | local | contact | Family | A.2.1 |
| 1402 | 14 | M | 74 | San Miguelito | 336023 | EPI_ISL_496834 | 2020-04-01 | 2020-04-03 | NA | unknown | local | contact | Family | A.2.1 |
| 1407 | 14 | F | 61 | Colón | 336044 | EPI_ISL_496835 | 2020-04-01 | 2020-04-03 | NA | unknown | local | contact | Family | A.3 |
| 1409 | 14 | M | 56 | Colón | 336047 | EPI_ISL_496836 | 2020-04-03 | 2020-04-03 | NA | unknown | local | unknown | NA | A.3 |
| 1418 | A | F | 25 | Panamá Metro | 336085 | EPI_ISL_496837 | 2020-04-03 | 2020-04-03 | NA | unknown | local | unknown | NA | A.2.1 |
| 1437 | 13 | F | 38 | Panamá Metro | 336231 | EPI_ISL_496838 | 2020-03-28 | 2020-04-04 | NA | unknown | local | contact | Family | A.2.1 |
| 1441 | 13 | M | 28 | Panamá Este | 336247 | EPI_ISL_496839 | 2020-03-25 | 2020-04-04 | NA | unknown | local | unknown | NA | A.2.1 |

| Case | Epi week | Sex | Age, y | Region | ID | GISAID Accession # | Date symptom onset | Date results received | Epi cluster | Epiclusterlink | Type of exposition | Epi link | Exposition description | Lineage autocolor |
|---|---|---|---|---|---|---|---|---|---|---|---|---|---|---|
| 1445 | 14 | F | 42 | Panamá Metro | 336264 | EPI_ISL_496840 | 2020-04-04 | 2020-04-04 | NA | unknown | local | contact | Family | B.1 |
| 1452 | 14 | M | 60 | San Miguelito | 336320 | EPI_ISL_496841 | 2020-03-31 | 2020-04-04 | NA | unknown | local | unknown | NA | A.2.1 |
| 1458 | 14 | M | 45 | Panamá Metro | 336341 | EPI_ISL_496842 | 2020-04-02 | 2020-04-04 | NA | unknown | local | unknown | NA | A.2.1 |
| 1460 | 14 | F | 40 | Panamá Norte | 336344 | EPI_ISL_496843 | 2020-04-02 | 2020-04-04 | NA | unknown | local | contact | Family | A.2 |
| 1466 | 14 | F | 15 | Panamá Oeste | 336377 | EPI_ISL_496844 | 2020-04-03 | 2020-04-04 | NA | unknown | local | contact | Family | A.2 |
| 1469 | 14 | M | 61 | San Miguelito | 336388 | EPI_ISL_496845 | NA | 2020-04-04 | NA | unknown | local | unknown | NA | A.2.1 |
| 1475 | 14 | F | 52 | Panamá Metro | 336416 | EPI_ISL_496846 | 2020-04-03 | 2020-04-04 | NA | unknown | local | contact | Family | A.2.1 |
| 1476 | 14 | M | 34 | San Miguelito | 336417 | EPI_ISL_496847 | 2020-04-04 | 2020-04-04 | NA | unknown | local | unknown | NA | A.2.1 |
| 1477 | 14 | F | 41 | Panamá Metro | 336418 | EPI_ISL_496848 | 2020-04-02 | 2020-04-04 | NA | unknown | local | unknown | NA | A.2.1 |
| 1480 | 14 | M | 48 | San Miguelito | 336432 | EPI_ISL_496849 | 2020-03-29 | 2020-04-04 | NA | unknown | local | unknown | NA | A.2.1 |
| 1481 | | F | 42 | Panamá Metro | 336440 | EPI_ISL_496850 | 2020-04-03 | 2020-04-05 | NA | unknown | local | unknown | NA | A.2.1 |
| 1484 | 13 | M | 32 | Panamá Este | 336491 | EPI_ISL_496851 | 2020-03-26 | 2020-04-05 | NA | unknown | local | unknown | NA | A.2.1 |
| 1490 | 14 | M | 33 | Panamá Metro | 336523 | EPI_ISL_496852 | 2020-04-04 | 2020-04-05 | NA | unknown | local | unknown | NA | A.2.1 |
| 1497 | 14 | F | 44 | Panamá Oeste | 336567 | EPI_ISL_496853 | 2020-04-03 | 2020-04-05 | NA | unknown | local | unknown | NA | A.2 |
| 1606 | 14 | F | 56 | San Miguelito | 336897 | EPI_ISL_496854 | 2020-04-01 | 2020-06-04 | NA | unknown | local | unknown | NA | A.2.1 |
| 1650 | 14 | M | 50 | Guna Yala | 337097 | EPI_ISL_496855 | 2020-04-04 | 2020-04-06 | NA | unknown | local | unknown | NA | A.2.1 |
| 1651 | 15 | M | 38 | Guna Yala | 337099 | EPI_ISL_496856 | 2020-04-05 | 2020-04-06 | NA | unknown | local | unknown | NA | A.2.1 |
| 1681 | 15 | M | 39 | Colón | 337250 | EPI_ISL_496857 | 2020-04-05 | 2020-04-07 | NA | unknown | local | unknown | NA | A.2.1 |
| 1689 | 14 | F | 26 | Los Santos | 337306 | EPI_ISL_496858 | 2020-04-04 | 2020-04-07 | NA | unknown | local | police | NA | A.2.1 |
| 1705 | 14 | F | 26 | Panamá Metro | 337358 | EPI_ISL_496859 | NA | 2020-04-07 | NA | unknown | local | contact | Family | A.2.1 |
| 1725 | 14 | M | 28 | Chiriqui | 337442 | EPI_ISL_496860 | 2020-04-04 | 2020-07-04 | NA | unknown | local | unknown | NA | A.2.1 |
| 1731 | 14 | M | 28 | Panamá Oeste | 337467 | EPI_ISL_496861 | 2020-04-05 | 2020-07-04 | NA | unknown | local | contact | Family | A.2.1 |
| 1775 | 14 | M | 27 | Chiriqui | 337640 | EPI_ISL_496862 | 2020-04-03 | 2020-07-04 | NA | unknown | local | unknown | NA | B.1.5 |
| 1778 | 15 | M | 43 | Panamá Oeste | 337660 | EPI_ISL_496863 | 2020-04-06 | 2020-07-04 | NA | unknown | local | unknown | NA | A.2.1 |
| 1781 | 14 | F | 55 | Panamá Oeste | 337668 | EPI_ISL_496864 | 2020-04-01 | 2020-07-04 | NA | unknown | local | unknown | NA | A.2 |
| 1834 | 15 | M | 36 | Panamá Oeste | 337884 | EPI_ISL_496865 | 2020-04-05 | 2020-04-08 | NA | unknown | local | unknown | NA | A.2.1 |
| 1895 | 14 | F | 46 | San Miguelito | 338258 | EPI_ISL_496866 | 2020-04-01 | 2020-04-08 | NA | unknown | local | unknown | NA | A.2.1 |
| 1904 | 15 | M | 23 | San Miguelito | 338362 | EPI_ISL_496867 | 2020-04-07 | 2020-04-09 | NA | unknown | local | police | NA | A.2.1 |
| 1910 | 15 | F | 25 | Panamá Metro | 338393 | EPI_ISL_496868 | 2020-04-05 | 2020-04-09 | NA | unknown | local | unknown | NA | A.2 |
| 1918 | 14 | M | 51 | Panamá Oeste | 338477 | EPI_ISL_496869 | 2020-04-02 | 2020-04-09 | NA | unknown | local | unknown | NA | A.2.1 |
| 1952 | 15 | F | 48 | Guna Yala | 338634 | EPI_ISL_496870 | 2020-04-06 | 2020-09-04 | NA | unknown | local | unknown | NA | A.2.1 |
| 1954 | 15 | M | 53 | Guna Yala | 338639 | EPI_ISL_496871 | 2020-04-06 | 2020-09-04 | NA | unknown | local | unknown | NA | A.2.1 |
| 1955 | 15 | F | 33 | Guna Yala | 338640 | EPI_ISL_496872 | 2020-04-06 | 2020-09-04 | NA | unknown | local | unknown | NA | A.2.1 |
| 1956 | 15 | F | 21 | Guna Yala | 338641 | EPI_ISL_496873 | 2020-04-06 | 2020-09-04 | NA | unknown | local | unknown | NA | A.2.1 |
| 1957 | 15 | M | 60 | Guna Yala | 338642 | EPI_ISL_496874 | 2020-04-06 | 2020-09-04 | NA | unknown | local | unknown | NA | A.2.1 |
| 1959 | 14 | M | 48 | Coclé | 338657 | EPI_ISL_496875 | 2020-04-02 | 2020-09-04 | NA | unknown | local | unknown | NA | B.1 |
| 1960 | 14 | M | 36 | Coclé | 338658 | EPI_ISL_496876 | 2020-03-29 | 2020-09-04 | NA | unknown | local | unknown | NA | B.1 |
| 1964 | A | M | 54 | Colón | 338674 | EPI_ISL_496877 | NA | 2020-09-04 | NA | unknown | local | contact | MP | B.1 |
| 1965 | A | M | 31 | Colón | 338677 | EPI_ISL_496878 | 2020-04-06 | 2020-09-04 | NA | unknown | local | unknown | NA | B.1 |
| 1966 | 15 | F | 30 | Coclé | 338681 | EPI_ISL_496879 | 2020-04-05 | 2020-09-04 | NA | unknown | local | contact | Family | A.2.1 |
| 1967 | 15 | M | 30 | Colón | 338687 | EPI_ISL_496880 | 2020-04-07 | 2020-09-04 | NA | unknown | local | contact | MP | B.1 |
| 1968 | 15 | M | 46 | Colón | 338689 | EPI_ISL_496881 | 2020-04-08 | 2020-09-04 | NA | unknown | local | contact | MP | B.1 |
| 1970 | 15 | F | 32 | Panamá Oeste | 338698 | EPI_ISL_496882 | NA | 2020-09-04 | NA | unknown | local | contact | Family | A.2.1 |
| 1972 | 15 | M | 32 | Coclé | 338706 | EPI_ISL_496883 | NA | 2020-09-04 | NA | unknown | local | police | NA | B.1 |
| 1981 | 15 | F | 25 | Colón | 338744 | EPI_ISL_496884 | 2020-04-08 | 2020-09-04 | NA | unknown | local | contact | Family | A.3 |
| 1984 | 14 | M | 70 | Panamá Metro | 338777 | EPI_ISL_496885 | NA | 2020-09-04 | NA | unknown | local | contact | Health | A.2.1 |
| 1993 | 15 | M | 47 | Panamá Metro | 338832 | EPI_ISL_496886 | NA | 2020-09-04 | NA | unknown | local | unknown | NA | A.2.1 |
| 2000 | 14 | M | 34 | Panamá Metro | 338859 | EPI_ISL_496887 | 2020-04-01 | 2020-10-04 | NA | unknown | local | unknown | NA | A.2.1 |

| Case | Epi week | Sex | Age, y | Region | ID | GISAID Accession # | Date symptom onset | Date results received | Epi cluster | Epiclusterlink | Type of exposition | Epi link | Exposition description | Lineage autocolor |
|---|---|---|---|---|---|---|---|---|---|---|---|---|---|---|
| 2004 | 14 | M | 55 | Panamá Metro | 338872 | EPI_ISL_496888 | 2020-04-03 | 2020-10-04 | NA | unknown | local | contact | Family | A.2.1 |
| 2024 | 15 | M | 21 | Panamá Norte | 338939 | EPI_ISL_496889 | 05/04/2020 | 2020-10-04 | NA | unknown | local | unknown | NA | A.2.1 |
| 2061 | 14 | F | 8 | Panamá Norte | 339064 | EPI_ISL_496890 | NA | 2020-04-10 | NA | unknown | local | unknown | NA | A.2.1 |
| 2074 | 15 | M | 27 | Coclé | 339125 | EPI_ISL_496891 | NA | 2020-04-10 | NA | unknown | local | unknown | NA | A.2.1 |
| 2083 | 15 | M | 28 | Colón | 339160 | EPI_ISL_496892 | NA | 2020-04-10 | NA | unknown | local | unknown | NA | A.3 |
| 2147 | A | M | 43 | Colón | 339459 | EPI_ISL_496893 | NA | 2020-04-11 | NA | unknown | local | unknown | NA | B.1 |
| 2148 | A | F | 57 | Panamá Oeste | 339467 | EPI_ISL_496894 | NA | 2020-04-11 | NA | unknown | local | unknown | NA | A.2.1 |
| 2152 | 15 | M | 22 | Colón | 339494 | EPI_ISL_496895 | NA | 2020-04-11 | NA | unknown | local | unknown | NA | A.3 |
| 2155 | 15 | M | 26 | Colón | 339499 | EPI_ISL_496896 | NA | 2020-04-11 | NA | unknown | local | unknown | NA | A.3 |
| 2165 |  | M | 35 | Coclé | 339631 | EPI_ISL_496897 | NA | 2020-04-12 | NA | unknown | local | unknown | NA | A.2.1 |
| 2166 | 15 | M | 40 | Colón | 339644 | EPI_ISL_496898 | NA | 2020-04-12 | NA | unknown | local | unknown | NA | A.3 |
| 2181 | 15 | M | 36 | Colón | 339755 | EPI_ISL_496899 | NA | 2020-04-12 | NA | unknown | local | unknown | NA | B.1 |
| 2182 | 14 | M | 38 | Colón | 339756 | EPI_ISL_496900 | NA | 2020-04-12 | NA | unknown | local | unknown | NA | B.1 |
| 2220 | 16 | M | 28 | Chiriqui | 340006 | EPI_ISL_496901 | 2020-04-12 | 2020-04-13 | NA | unknown | local | unknown | NA | A.2.1 |
| 2244 | 15 | F | 47 | Panamá Oeste | 340172 | EPI_ISL_496902 | 2020-04-08 | 2020-04-13 | NA | unknown | local | unknown | NA | A.2 |
| 2266 | 15 | F | 23 | Guna Yala | 340229 | EPI_ISL_496903 | 2020-04-09 | 2020-04-13 | NA | unknown | local | unknown | NA | A.2.1 |
| 2267 | 15 | M | 68 | Guna Yala | 340232 | EPI_ISL_496904 | 2020-04-09 | 2020-04-13 | NA | unknown | local | unknown | NA | A.2.1 |
| 2269 | 15 | F | 22 | Guna Yala | 340239 | EPI_ISL_496905 | 2020-04-10 | 2020-04-13 | NA | unknown | local | unknown | NA | A.2.1 |
| 2367 | 16 | M | 31 | Panamá Oeste | 340836 | EPI_ISL_496906 | NA | 2020-04-14 | NA | unknown | local | unknown | NA | A.2.1 |
| 2368 | 15 | F | 41 | Panamá Oeste | 340837 | EPI_ISL_496907 | 2020-04-10 | 2020-04-14 | NA | unknown | local | unknown | NA | B.1 |
| 2414 | A | M | 46 | Colón | 341001 | EPI_ISL_496908 | NA | 2020-04-14 | NA | unknown | local | unknown | NA | B.1 |
| 2415 | A | M | 37 | Colón | 341003 | EPI_ISL_496909 | NA | 2020-04-14 | NA | unknown | local | unknown | NA | B.1 |
| 2418 | 15 | M | 34 | Colón | 341008 | EPI_ISL_496910 | 2020-04-11 | 2020-04-14 | NA | unknown | local | unknown | NA | B.1 |
| 2419 | A | M | 42 | Colón | 341009 | EPI_ISL_496911 | NA | 2020-04-14 | NA | unknown | local | unknown | NA | B.1 |
| 2422 | 15 | M | 29 | Bocas Del Toro | 341042 | EPI_ISL_496912 | 2020-04-11 | 2020-04-14 | NA | unknown | local | unknown | NA | A.3 |
| 2463 | 16 | M | 21 | San Miguelito | 341259 | EPI_ISL_496913 | 2020-04-12 | 2020-04-15 | NA | unknown | local | unknown | NA | A.2.1 |
| 2466 | 16 | M | 26 | Guna Yala | 341269 | EPI_ISL_496914 | 2020-04-13 | 2020-04-15 | NA | unknown | local | unknown | NA | A.2.1 |
| 2481 | 16 | M | 22 | Colón | 341361 | EPI_ISL_496915 | NA | 2020-04-15 | NA | unknown | local | unknown | NA | A.2.1 |

epi, epidemiological; NA, not available.

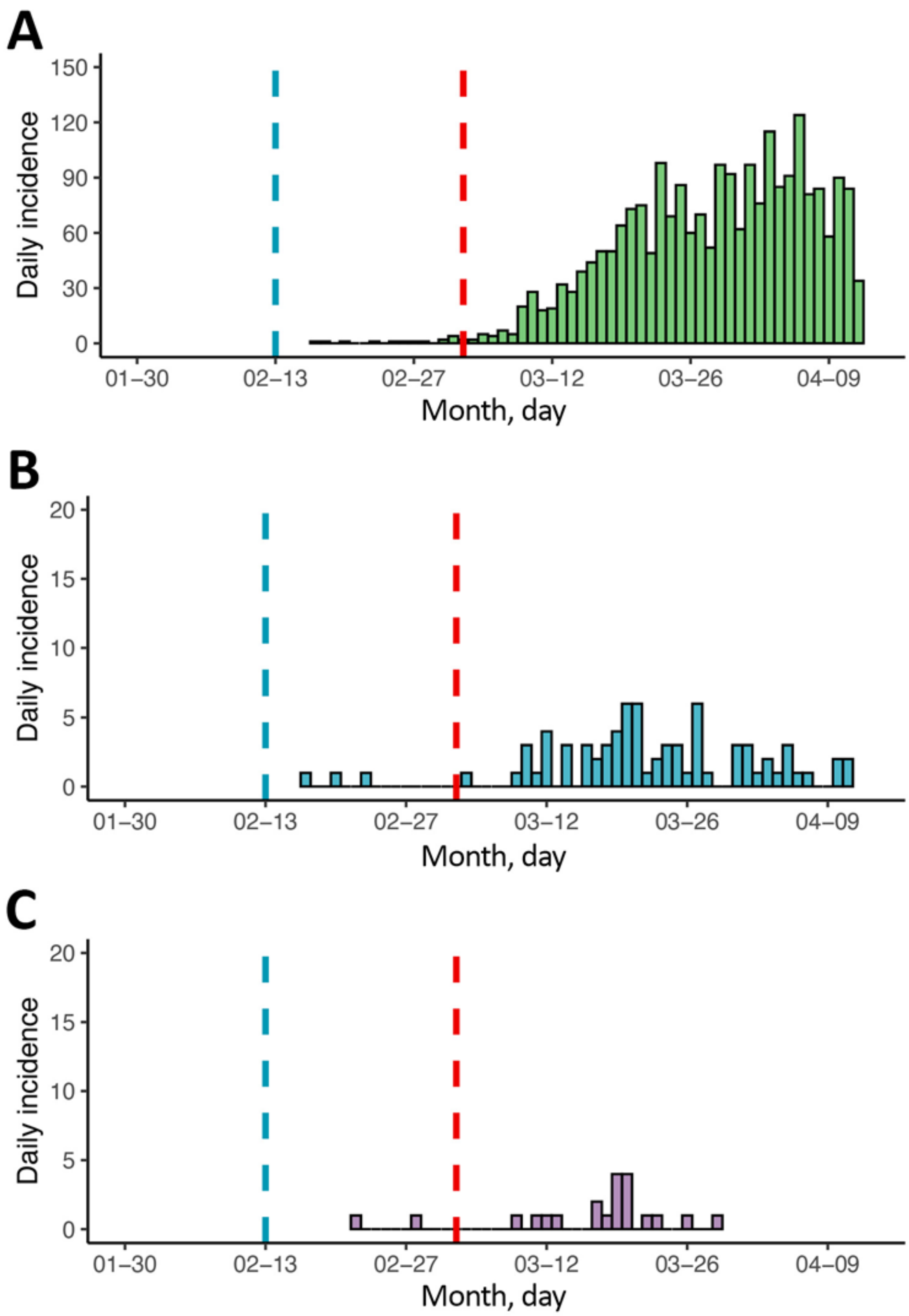


**Appendix 2 Figure 1.** Daily incidence of SARS-CoV-2 cases in Panama: A) ambulatory patients (asymptomatic, pre-symptomatic, mildly symptomatic outpatient), B) hospitalized patients, and C) patients who had died, detected through April 16, with symptom onset during February 15–April 13. In all 3 charts, the y-axis represents the daily incidence and the x-axis represents the date of symptom onset for each reported case. The blue dashed vertical lines represent the first recorded onset of symptoms and red dashed vertical lines the date of first case confirmed by the surveillance system.

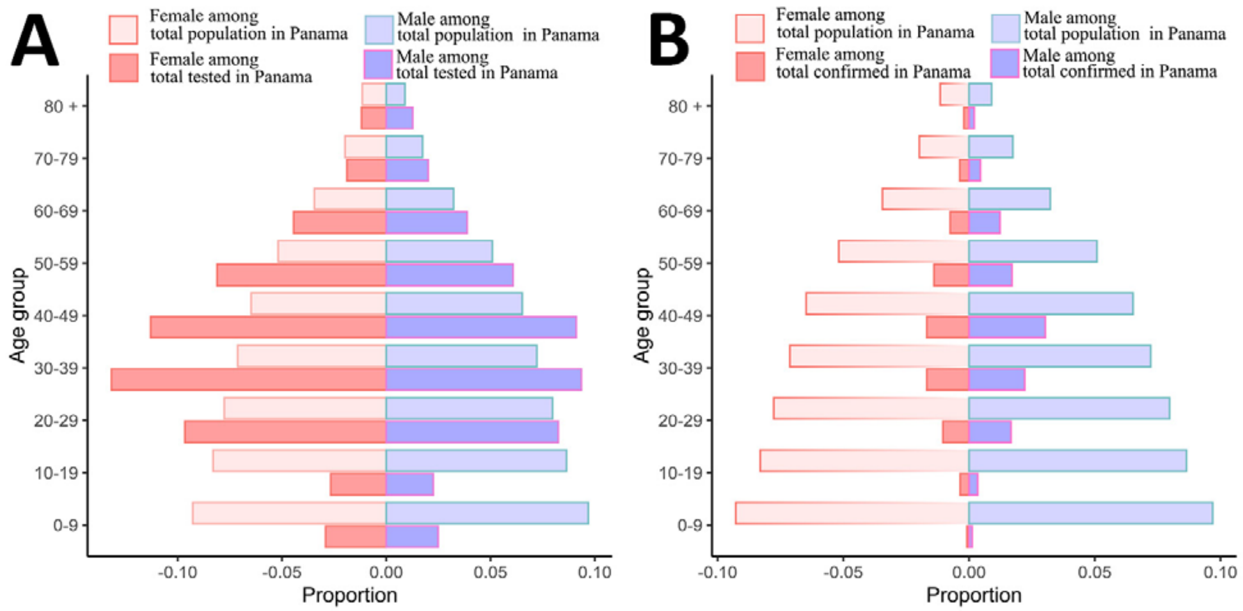


**Appendix 2 Figure 2.** Age and sex distribution of tested subjects in Panama. Proportion of subjects with age group distribution and sex proportion of A) tested and B) SARS-CoV-2–confirmed cases.

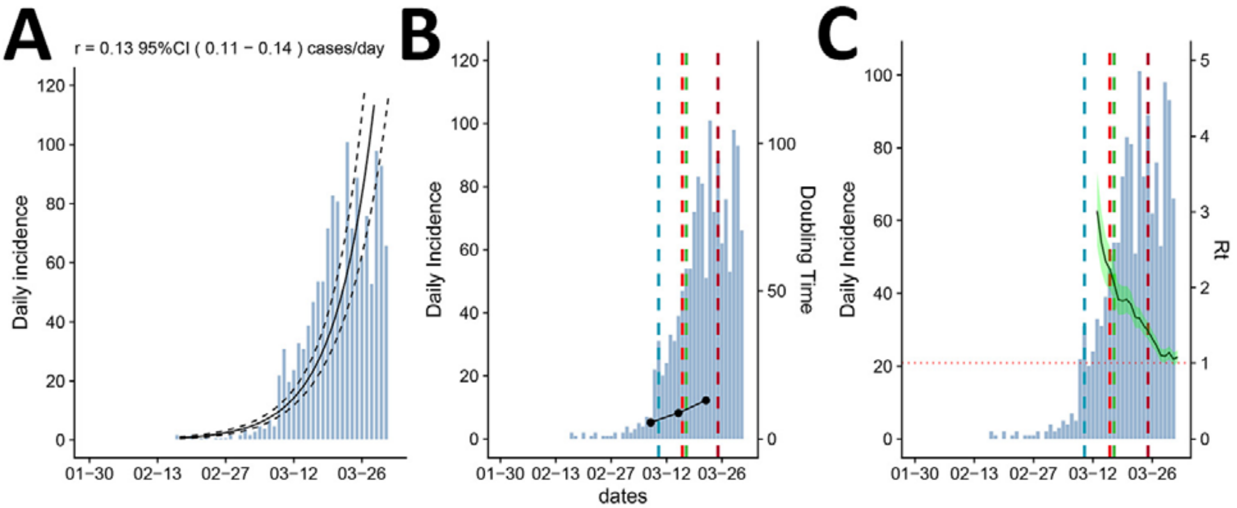


**Appendix 2 Figure 3.** Estimates of epidemiologic dynamics in Panama. Number of COVID-19 daily confirmed infections (left axes and bars) overlaid with estimates of A) fitted exponential growth in cases daily growth rate; B) doubling time; and C) time-varying effective reproduction number $R_t$, for a time frame of 45 days (x-axes). For C), green shaded areas show 95% confidence intervals around the median estimated $R_t$. The threshold value $R_t = 1$ is indicated by the red dashed horizontal line. For both B) and C), dashed vertical lines indicate the implementation dates of school closures (blue), night curfews (red), restrictions of movement (green), and the 24-hour curfew (purple).

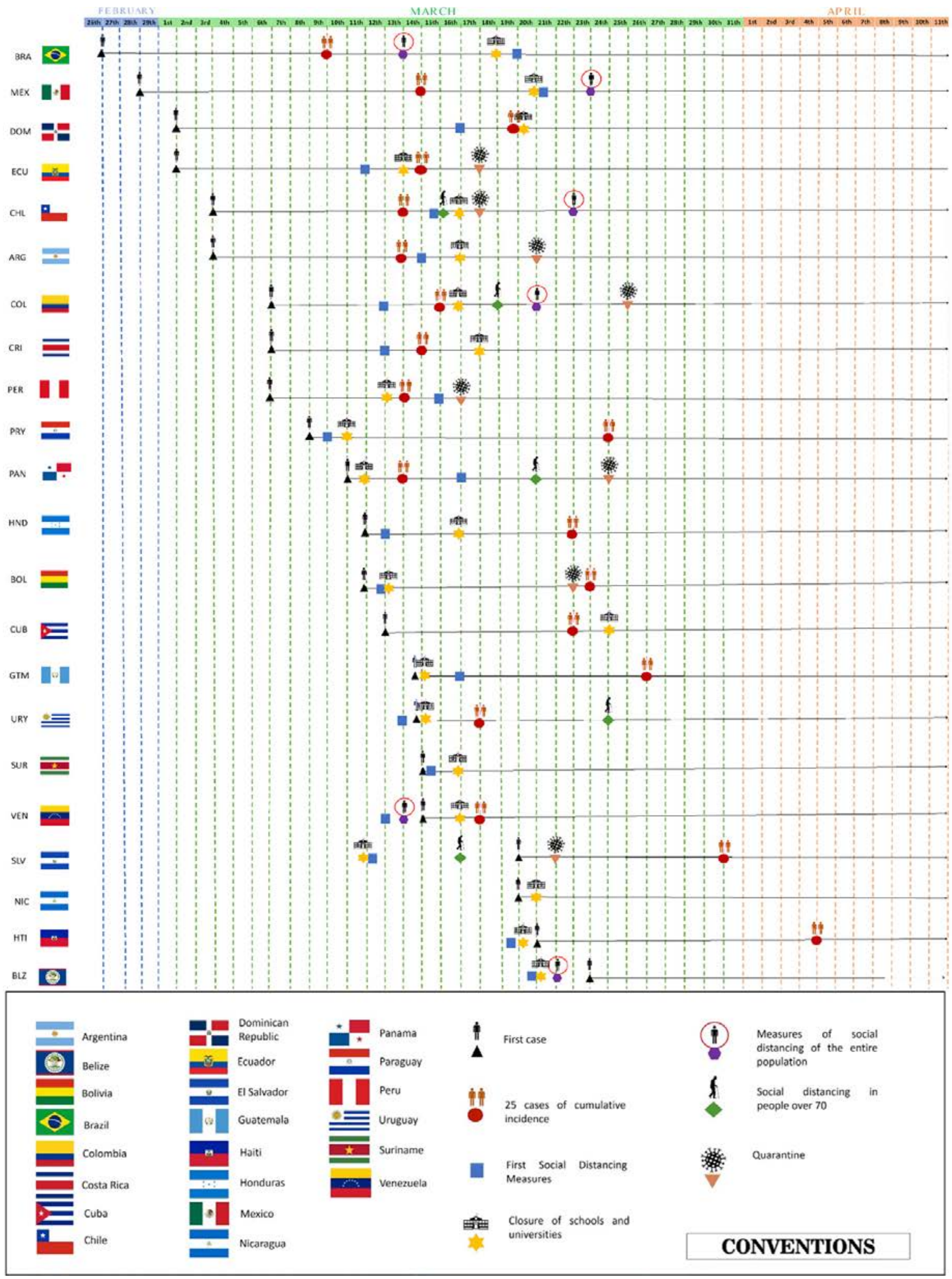


**Appendix 2 Figure 4.** SARS-CoV-2 timelime in Latin America. Timeline of the first reported SARS-CoV-2 infection and time frame to adopt control strategies for each Latin American country based on official report of Panama MoH and media reports.

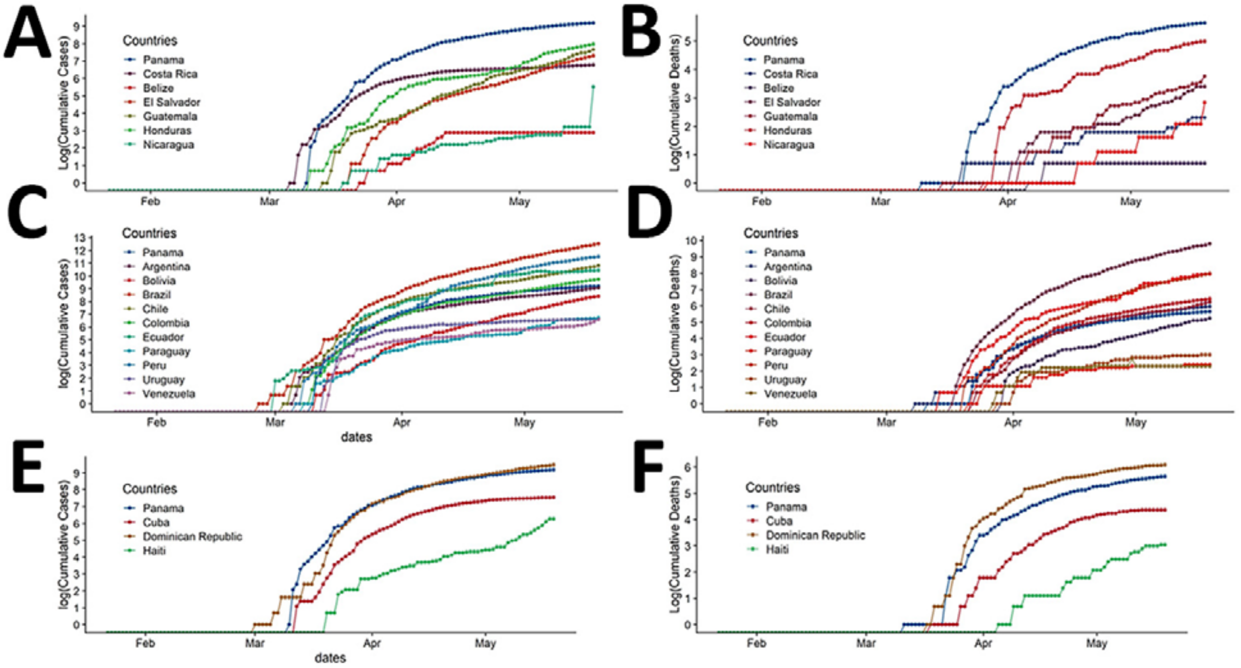


**Appendix 2 Figure 5.** Cumulative incidence and deaths over time in Panama compared with A) Central America (incidence), B) Central America (deaths), C) South America (incidence), D) South America (deaths), E) Caribbean countries (incidence), and F) Caribbean countries (deaths).

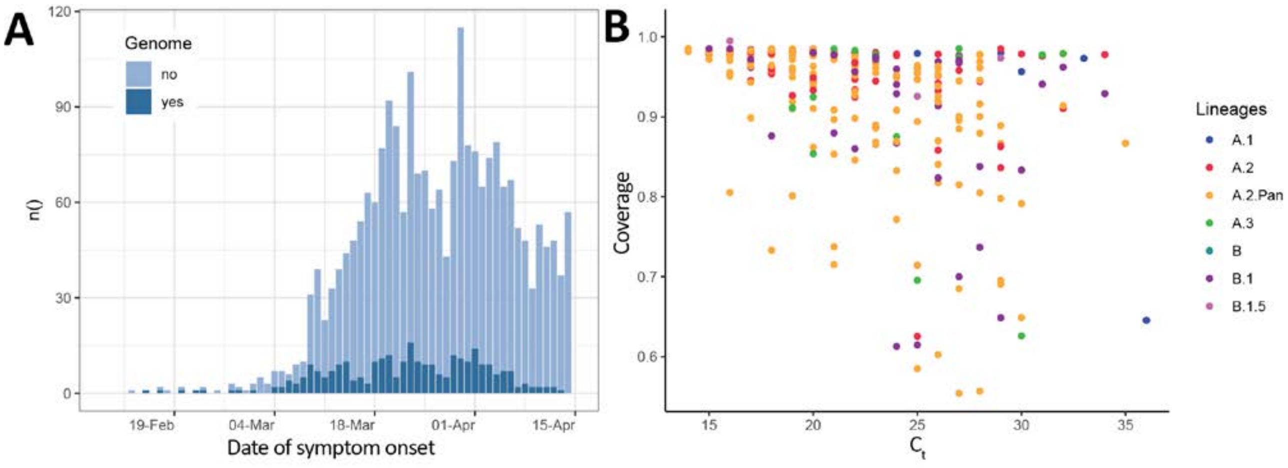


**Appendix 2 Figure 6.** Daily distribution and characteristics of SARS-CoV-2 genomes obtained in the study. A) COVID-19–confirmed cases and SARS-CoV-2 genomes distribution during the analyzed period in Panama, by date of symptom onset reported by the patient. B) Plot of proportion of genome coverage versus $C_t$ value obtained in real-time PCR, dots are colored according to lineages.

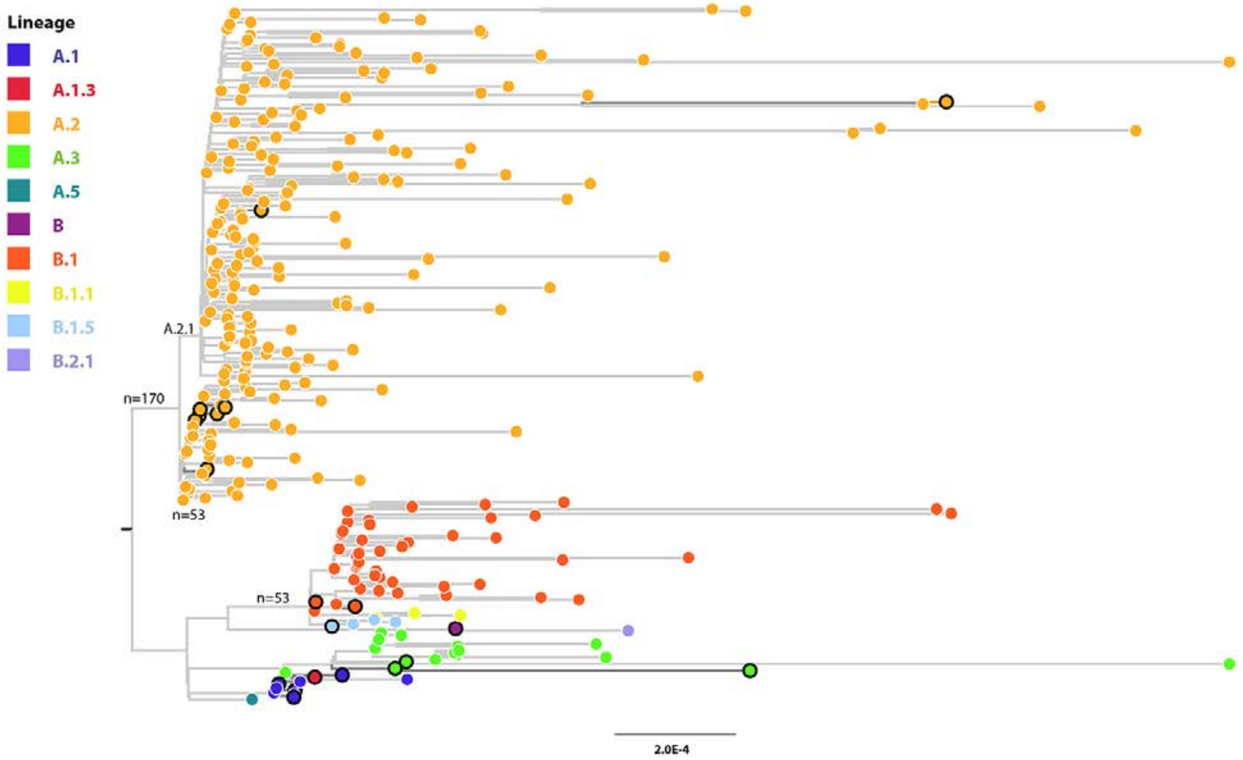


**Appendix 2 Figure 7.** Maximum likelihood tree of the SARS-CoV-2 genomes obtained (n = 313) circulating in Panama. Tip shapes were colored according to the inferred lineage of the samples. Circles outlined in black indicate samples with travel-related epidemiologic link.

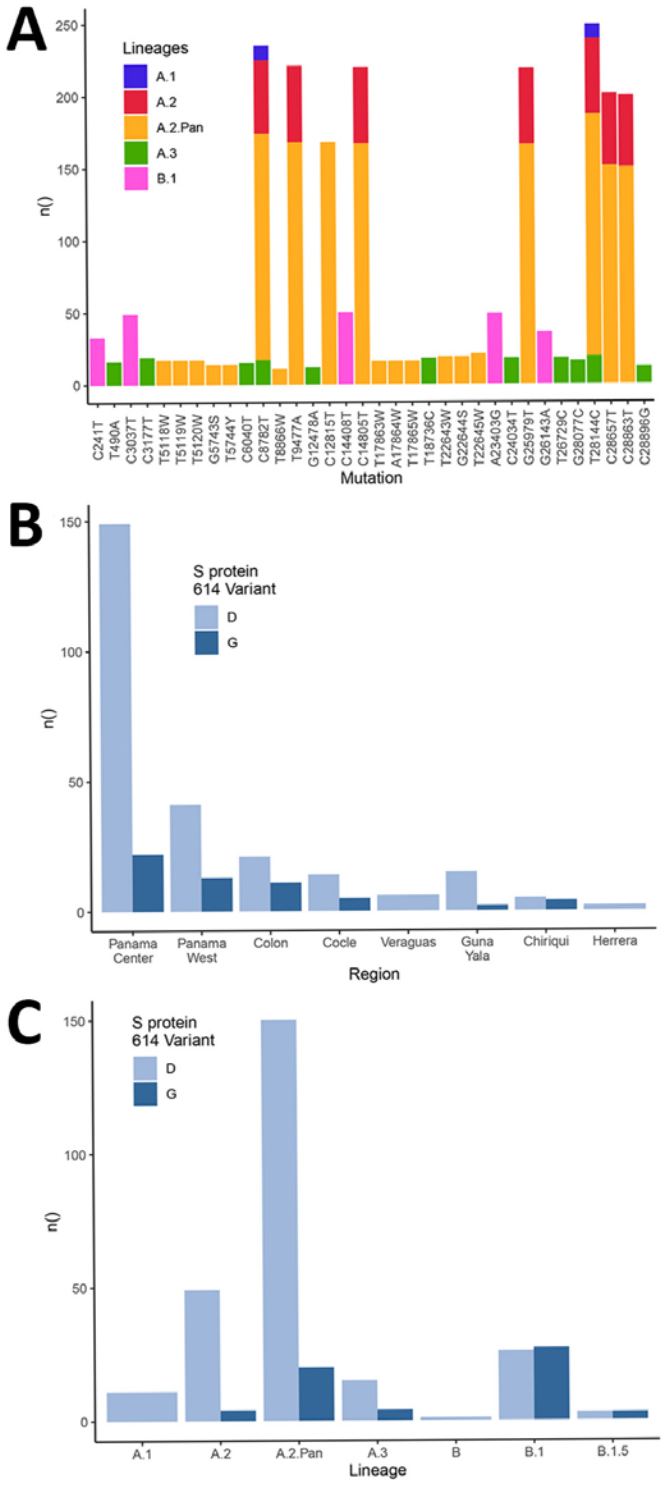


**Appendix 2 Figure 8.** Mutation profile of the sequences obtained in the study: A) frequency of single nucleotide polymorphism in the genome (position according to MN908947) for all analyzed sequences (n = 313) in the study. Distribution of S protein variants, D614 or G614, B) in different regions of the country or C) among lineages.
